# Randomized studies in China show that studying and promoting civic honesty needs to consider local norms

**DOI:** 10.1038/s41598-025-87804-z

**Published:** 2025-01-29

**Authors:** Iris W. Hung, Yuho Yiu, Sihan Wu, Dan Liu, Liman Wang, Xiao Han

**Affiliations:** 1 Department of Marketing, The Chinese University of Hong Kong, Shenzhen, China; 2 Department of Computer Science and Engineering, Hong Kong University of Science and Technology, Hong Kong, China; 3 Department of Marketing, Tsinghua University, Beijing, China; 4 Department of Marketing, Fudan University, Shanghai, China; 5 Hangzhou Fancha Technology, Hangzhou, China

**Keywords:** Psychology, Human behaviour

## Abstract

Civic honesty is a crucial behavior that policymakers and business leaders strive to promote among citizens and employees. We conducted online and field experiments in China adapting the setting of a recent research which employed an innovative design to study civic honesty behavior around the globe. Results show that the psychological mechanism underlying lost-wallet reporting behavior in China is somewhat different from what has been suggested in the literature. Chinese participants indicated they are significantly more likely to put WeChat than email as contact information on their lost items, corroborating a local norm in China that email is infrequently used by ordinary Chinese citizens. Moreover, we tested multiple behavioral interventions in the field. Aiding a lost-property finder to connect to an owner using WeChat led to a reporting rate of 59.3% (vs. 7% as shown in a recent research that used email as the communication means; note that due to differences in experimental designs, no direct comparison of these reporting rates should be made however). Our research suggests that interacting with citizens from a targeted population to formulate locally informed research questions and design may be crucial for understanding and promoting civic honesty behavior.

## Introduction

Civic honesty is crucial for the prosperity and development of societies. Political fraud, corruption, corporate scandal, and tax evasion could cause substantial economic losses, erode trust in societies and ethics in organizations, and eventually bring immense psychological pain to individuals^1–5^. Understanding the issue, including why people engage in honest behavior, how to foster civic honesty, and the policy implications is important. To understand civic honesty across countries, Cohn et al.^6^ (hereafter Cohn) used an innovative setting in which lost wallets were turned in across 40 countries and people’s reporting rates of these lost wallets were measured. Reporting a lost wallet is a proxy for civic honesty behavior. In their field experiment, incentives for dishonesty were operationalized by varying the amount of money and the presence of personal items in the wallets. Results revealed that across countries, recipients were more likely to report a lost wallet when it contained personal items (vs. not) or when it contained money (vs. not). Cohn further investigated the psychological mechanism using samples from the US, the UK, and Poland and showed that participants’ intention of returning a lost wallet can be jointly driven by two psychological factors: altruistic concern and theft aversion. The primary goal of the current research is to identify effective ways to promote civic honesty behavior in China. We consider China because in Cohn, the lost-property reporting rate is the lowest in the world (7% when a lost-property did not contain money but only personal items). Our field and lab experiments adapted Cohn’s experimental design and showed that civic responsibility, altruistic concern, theft aversion, and scam suspicion independently motivate lost-wallet reporting behavior. Moreover, Chinese participants indicated that they are more likely to put WeChat than email as the contact information they put on their lost-items (i.e., owners can be contacted when the lost-items are found and reported). Our field experiment showed an average reporting rate of 59.3%. Our research design is adapted and different from Cohn in multiple ways and therefore no direct comparisons of the present research and Cohn should be made. In sum, our studies suggest that considering local norms may be important for studying and improving civic honesty behavior.

## Factors influencing and interventions for motivating civic honesty behavior

Past research using approaches including cognitive and developmental, personality traits, impression management concerns, methodological, and group influences have documented aspects that make people behave dishonestly^7–15^. For example, people are willing to forego up to three-quarters of their gains from being dishonest. Group dishonesty exacerbates with an increase in joint profits. Other factors, including, experimental settings (e.g., contexts, incentives for cheating), the extent to which one is being trusted, concern about self-image, and the preference to be perceived as honest motivate honesty. Research on factors influencing civic honesty behavior has been particularly few. One exception is the work of Cohn, which conducted a large-scale field study in 355 cities across 40 countries. In their study, over 17,000 wallets were “lost.” The amount of money and the presence of personal items contained in these lost wallets were varied. Reporting rates across countries range from 7 to 76%. In particular, China has the lowest reporting rate. In a follow-up survey conducted in three countries, US, UK, and Poland, the authors showed that the presence of money is related to an aversion of being seen as a thief when people fail to report a lost wallet whereas the presence of personal items is related to altruistic concern.

Many of these abovementioned studies were conducted in the lab. Field experiments investigating factors influencing honesty and testing interventions for motivating honesty have been relatively few^16^. Building on the existing research, we intend to test and design interventions for motivating civic honesty behavior in China. To do this, we adapted the innovative design of Cohn’s online and field studies. Our online studies showed that civic responsibility, altruistic concern, theft aversion, and scam suspicion significantly influences Chinese citizens’ lost-wallet reporting behavior. In a field study conducted in China, we tested effects of three types of interventions against a no-intervention condition (control): civic responsibility, altruistic concern, and safety assurance.

We develop our hypotheses based on (1) related findings in the literature and (2) our observations in online discussions. We hypothesize that civic responsibility, altruistic concern, and safety assurance interventions may independently increase wallet reporting rate. First, legal and moral reminders are often tested as nudges in research. The effects of reminding participants of the honor system, the legal consequences of being dishonest, and the honor code have been studied in contexts such as non-proctored exams, online exams, and self-service newspaper stalls^17–20^. Mixed results have been yielded^21,22^. From our informal interviews and observations in online discussion, local people mentioned that there has been civic education communication (in the form of a song) which educates citizens to report lost-property. In the current research, we examine whether reminding citizens of their responsibility of reporting lost property may dispose them to engage in lost wallet reporting behavior. Second, based on the findings in Cohn, we developed and tested interventions that elicit altruistic concern and theft aversion of citizens. Numerous research has shown that prompting people to take other person’s perspective by imagining the plight of others could evoke altruistic motives and behavior^23,24^. Thus, an intervention putting people to consider how the owner would feel about losing a precious personal item should activate altruistic motives and motivate reporting behavior. Past research has demonstrated the effect of self-image on honesty. The more people attend to how they are evaluated by others or portraying a positive self-image, the more likely they engage in honesty behavior^4^. Third, according to the results of Online Experiment 1 and our observation in online discussions, it is plausible that people may feel suspicious that a lost wallet is a scam. For example, in online discussions, citizens mentioned that “it is plausible that an owner may claim that the wallet contained a large amount of money—indicating that the money is gone and stolen by the finder.” Indeed, similar scams had happened in the population. This may serve as a cue for activating suspicion of ulterior motives of a lost-item owner^25^. Research showed that people generally believe information about others’ behavior at face value. Once the cues for suspicion are activated, people may become skeptical of ulterior motives (e.g., scam) of others. We therefore assume that assuring the safety of reporting a lost wallet (i.e., it is not a scam) may help clear suspicions and motivate reporting. However, it is also plausible that such a safety assurance intervention may ironically exacerbate suspicion or safety concern. Consequently, such an increase in the salience of safety concern may decrease reporting.

Finally, to further enhance the potential impact of these interventions, we considered a communication means that is commonly used in China. Past research has shown that choice-architectural interventions that can lower psychological frictions of engaging in good behavior may be helpful^26^. Common architectural intervention design include default automatic enrolment, mandatory choice requirement, and nudges for lowering psychological frictions that make a desired behavior difficult. We conjecture that if citizens use a reliable communication means that they habitually use and can be easily initiated (e.g., opened by just scanning a QR code), such a means could lessen these frictions or overcome any cultural habits (e.g., preferring to safekeep a lost property^27–32^) that hinder people from reporting a lost wallet. Based on our local knowledge and interactions with China samples, a local norm is that email is seldom used by ordinary citizens^33–35^. Thus, instead of using email, we use WeChat, a social networking app that is frequently used by Chinese citizens (citizens aged from 16 to 64 interact with WeChat frequently every day^36^) as the communication means between a lost-wallet finder and an owner^37,38^. This applies to all our treatment conditions in our online and field experiments.

## Considering local norms may be crucial

Many of the existing studies on honesty mostly considered populations from the so-called WEIRD (Western, Educated, Industrialized, Rich, and Democratic) samples. Psychologists have recently urged researchers to consider local norms and cultural characteristics in order to understand diverse phenomena and promote good behavior across different populations. Researchers have recently called for expanding interpretive power of behavioral science by not assuming that any psychological processes identified from any particular group or context are generalizable to all cultures^39–44^. In the context of civic honesty behavior, this may challenge an assumption that the psychological mechanisms identified in the literature are representative of non-WEIRD societies. As William McGuire argued, it is important to consider the groups from whom research findings derive and how researchers’ own cultural experiences shape their predictions and assumptions^45–48^. Relatedly, a recent study showed that cultural orientation is significantly correlated with citizens’ lost-wallet reporting behavior across countries documented in Cohn^27^. In particular, collectivism is negatively correlated with wallet-reporting behavior. These discussions and findings combine to suggest that it may be important to consider cultural characteristics and local norms when studying and improving civic honesty behavior in different populations.

Via interacting with China samples, our ultimate goal is to use more culturally informed research questions to improve our understanding and ability to foster civic honesty in our target population^9^. To accomplish our goal, we first conducted online searches and informal interviews with local people on factors motivating people to report a lost property. Our observations are that (1) theft aversion, civic responsibility, altruistic concerns, and scam suspicions may drive lost-property reporting behavior, and (2) local norms in China that WeChat is commonly used whereas email is infrequently used by ordinary citizens in China are related to the present context of interest. To verify the presence of the latter local norms in the context of lost-and-found, we conducted a preliminary study. Chinese participants indicated that they are significantly more likely to use WeChat than email as contact information for their lost items (M_WeChat_ = 5.061 vs. M_email_ = 3.561; *P* < 0.00001; on a 7-point scale from 1 = not at all to 7 = very likely). They also indicated that using WeChat as a contact information for their lost items is a realistic and standard practice (M_standard practice_ = 5.052 vs. midpoint = 4.000; t(263) = 12.94, *P* < 0.0001; on 7-point scales), and putting a QR code on everyday objects is commonplace (M = 5.019 vs. midpoint; t(263) = 10.21, *P* < 0.0001) in China.

Based on these preliminary findings, we developed our studies to (1) investigate the psychological mechanism underlying civic honesty behavior, and (2) test effects of multiple interventions. The civic responsibility intervention message reminds people of their responsibility of returning lost property. The altruistic concern intervention message elicits empathy by mentioning that the item can be precious to the owner (i.e., do not assume that an item is worthless and ignore it). The safety assurance intervention message attempts to assure people that the lost item is not a scam (Table 3). Online Experiment 1 examined whether theft aversion, civic responsibility, altruistic concerns, and scam suspicion may drive lost wallet reporting behavior. Online Experiment 2 examined the effects of our interventions and the psychological mechanism underlying these effects. Lastly, a field experiment tested our interventions in the field.

## Results

**Fig. 1:**
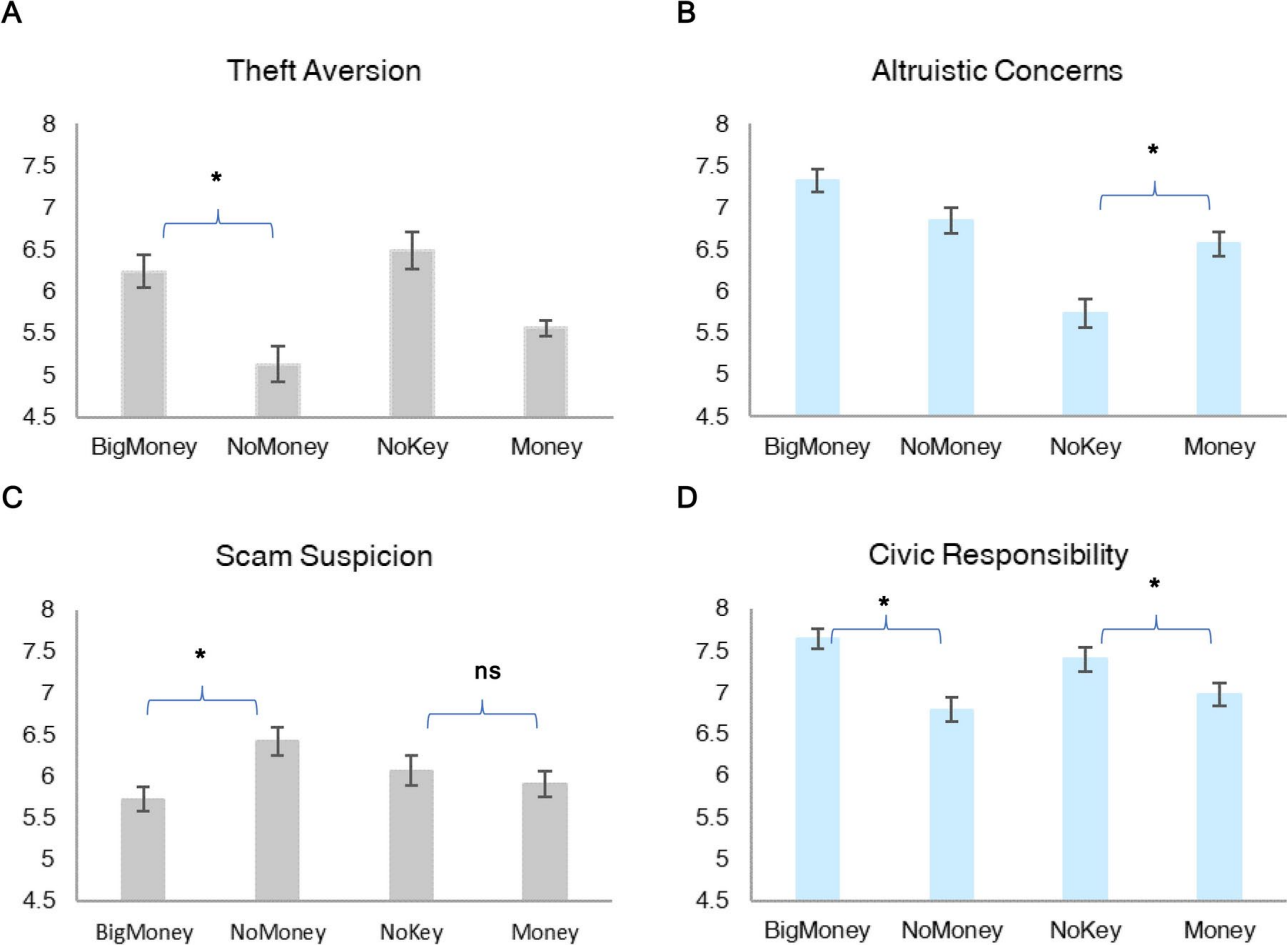
Theft aversion, altruistic concerns, scam suspicion, and civic responsibility as a function of wallet content conditions in Online Experiment 1 (N = 1,052). The vertical axes of (**A**–**D**) are the intensity of the four psychological factors (1 = not at all; 11 = very much so). Sample sizes in each experimental condition (N_BigMoney_ = 246; N_NoMoney_ = 231; N_NoKey_ = 235; N_Money_ = 248). Error bars represent standard error of the mean. *, the hypothesized effect of wallet content (the presence of money, or the presence of personal items) on the psychological factor is statistically significant. ns, not statistically significant.

**Fig. 2:**
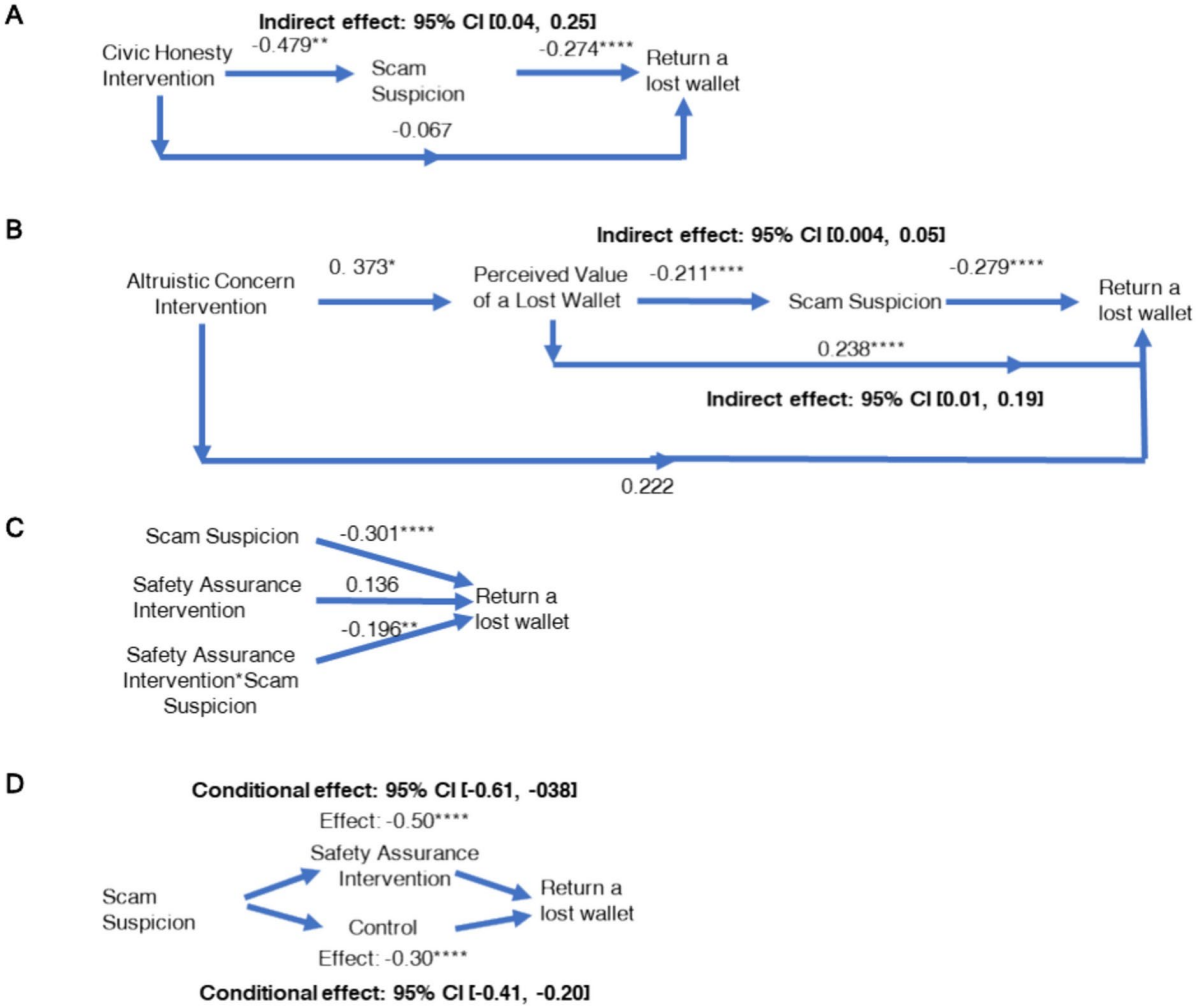
Path analyses of the effect of interventions in Online Experiment 2 (N = 1496). (**A**) The indirect effect of the civic honesty intervention via scam suspicion (n = 760), comparing the civic honesty intervention and the control condition. (**B**) The indirect effect of the altruistic concern intervention via perceived value of a lost wallet and then perceived scam suspicion (n = 748), comparing the altruistic concern intervention and the control condition. (**C**) and (**D**) The moderating effect of the safety assurance intervention, the conditional effect of the presence of safety assurance intervention (n = 740), comparing the safety assurance intervention and the control condition. The numbers reported are coefficient estimates and 95% confidence intervals (CI). Significance level: * *P* < 0.05, ** *P* < 0.01, *** *P* < 0.001, and **** *P* < 0.0001.

**Fig. 3:**
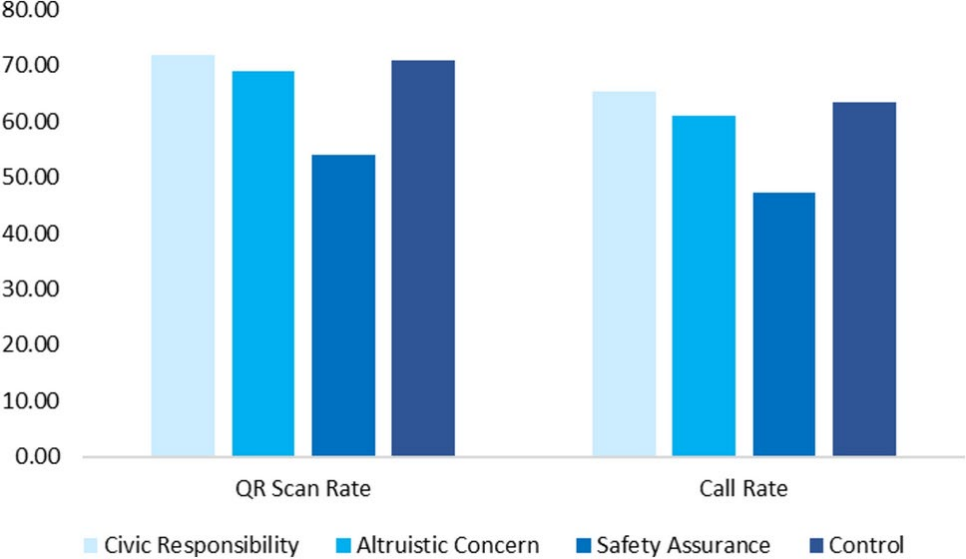
Wallet reporting rate (%) in terms of QR-scan rate and phone call rate. QR Scan (to open a WeChat page) and phone call (made on a WeChat page) rates as a function of interventions in Field Experiment (all wallets contained no money and just personal items). Sample sizes in each experimental condition (N_CivicResponsibility_ = 162; N_AltruisticConcern_ = 167; N_SafetyAssurance_ = 165; N_Control_ = 164).

The research was approved by the Institutional Review Board of the Chinese University of Hong Kong, Shenzhen and Tsinghua University. All methods were performed in accordance with the relevant guidelines and regulations. Informed consent was obtained from participants prior to participating in the online experiments. Figures 1 and 2 show the psychological mechanism examined in Online Experiments 1 and 2. Figure 3 shows the lost wallet reporting rate across intervention conditions in the field experiment. We describe the methods, materials, and results in detail in Supplementary Information.

### Online experiment 1

In this study, our main objectives were to (1) examine whether and how wallet content influences people’s behavioral intention of reporting a lost wallet, and (2) whether theft aversion, civic responsibility, altruistic concern, and scam suspicion play a role in these effects. Participants (*N* = 1052) were Chinese citizens from eight cities (Beijing, Chengdu, Guangzhou, Hangzhou, Shanghai, Shenzhen, Tianjin, and Xi’an). We varied the content in a wallet and employed a one-factor, four-level (BigMoney vs. Money vs. NoMoney vs. NoKey) experimental design. Our data showed that the presence of personal items (i.e., a key) elicits altruistic concern (*P* < 0.00001) whereas the presence of money intensifies participants’ fear of being seen as a thief if they fail to return a lost wallet (*P* < 0.001). Altruistic concern and theft aversion significantly mediated the effect of wallet content on participants’ intention of reporting a lost wallet. More importantly, beyond these effects, we also found that scam suspicion and civic responsibility significantly varied as a function of wallet content and mediated the effect of wallet content on reporting behavior. Participants felt a stronger sense of civic responsibility when the wallet contained a large amount of money than when it contained no money (*P* = 0.00002). Participants had a significantly stronger feeling of scam suspicion when a wallet did *not* contain any money than when it contained a large amount of money (*P* = 0.002; Fig. 1).

### Online experiment 2

Online Experiment 1 revealed that the psychological mechanism underlying Chinese citizens’ wallet reporting behavior was somewhat different from what has been suggested in the literature. Based on these results, we developed and tested the impact of several behavioral interventions in Online Experiment 2; Table 1). A total of 1670 participants from over twenty cities in China were randomly assigned to one of the eight intervention conditions of a 4 (interventions: civic responsibility vs. altruistic concern vs. safety assurance vs. control) × 2 (the presence of money: Money vs. NoMoney) between-subjects design. We adapted the experimental design in Cohn’s experiment with three aspects of differences. As in Online Experiment 1, we used WeChat as the communication means and measured theft aversion, altruistic concerns, scam suspicion and civic responsibility. In addition, we tested the effects of four versions of intervention messages (see Table 3 for the intervention messages, Methods and *SI*, Fig. S2). Results showed that the civic responsibility intervention significantly lowered scam suspicion (*P* = 0.007) compared to the control (i.e., no intervention) condition. Consequently, the lower the scam suspicion, the higher the wallet reporting likelihood. Scam suspicion significantly mediated the impact of the civic responsibility intervention on the wallet reporting likelihood. The altruistic concern intervention significantly increased participants’ perception of the extent to which the wallet was precious to the owner (*P* = 0.029) and directionally lowered scam suspicion (*P* = 0.059) compared to the control condition. Both altruistic concern and scam suspicion mediated the impact of altruistic intervention on reporting likelihood (See Fig. 2 for path analyses). The safety assurance intervention did not significantly lower scam suspicion, compared to the control condition (*F* < 1). Moreover, it significantly decreased participants’ intention of returning the lost wallet (*P* < 0.0001), compared to the control condition. We expected that assuring the safety of contacting the owner would lower scam suspicion and motivate reporting. Results suggest that this may backfire and increase the salience of safety concerns which may ironically exacerbate its negative impact on the stated likelihood of reporting a wallet. This corroborates our assumption that people are suspicious about the lost property being a scam. To the extent that this concern is made salient, people’s reliance on this concern increases and their motivation to return the wallet could be dampened. To assess the validity of this latter assumption, the wallet reporting intention (as dependent variable), the presence of safety assurance intervention, participants’ perceived suspicion, and their interaction terms (as independent variables) were considered in a moderation analysis^49^. Results supported our prediction: the presence of safety assurance intervention significantly moderated the impact of perceived scam suspicion on reporting intention (interaction: β = − 0.196, SE = 0.08, *P* = 0.013). In particular, the presence of safety assurance intervention (vs. control) significantly *enhanced* the negative impact of perceived scam suspicion on wallet reporting intention (β = − 0.50, SE = 0.06, *P* = 0.0000, 95% CI [− 0.61, − 0.38]; see Fig. 2 for path analyses).

**Table 1:** Key features and adaptations of study designs in Cohn and the current research.

	Cohn	The current research
Online experiment 1		
Samples	The US, The UK, and Poland	China
Number of samples	2,525 (n = 829 in the UK, n = 809 in Poland, and n = 887 in the US)	1,052
Lost-wallet scenario	Same	Same
Psychological factors	Altruistic concerns, theft aversion	Altruistic concerns, theft aversion; Additional measures on scam suspicion and civic responsibility
Civic honesty measurement	Reporting intention	Reporting intention
Online experiment 2		
Samples	N/A	Over twenty cities in China
Number of samples	N/A	1,670
Interventions	N/A	Four behavioral interventions
Field experiment		
Samples	Eight cities in China	Two most populous cities in China (Shanghai and Beijing)
Number of samples	400	660
Interventions	N/A	Four behavioral interventions
Wallet drop-off procedure	Same	Same
Drop-off locations	Societal institutions	Societal institutions
Civic honesty measurement	Email response	Phone call initiated on Wechat
Wallet	Transparent in color	Transparent in color
Contact means	Email on a business card	WeChat on a QR code sticker

### Field experiment

A preregistered (https://aspredicted.org/3cnv-4vy9.pdf) field experiment was run in two most populous cities in China, Shanghai and Beijing. We turned in 660 wallets. They were dropped off in four types of societal institutions: (1) pharmacies; (2) museums, theaters, libraries, or other cultural establishments; (3) hotels; and (4) police stations. All of these institutions have a reception area where the research confederates could turn in the wallets.

We adapted the experimental design of a field study in Cohn (Table 1 for key features and adaptations). All lost wallets in our experiment contained a key (i.e., no money). We focused on lost wallet that do not contain money (as noted earlier, the reporting rate was 7% as shown in Cohn, when a wallet did not contain money). Instead of using unique email address for each wallet to record whether the recipient received a particular wallet contacted the owner, we put a unique QR code (In China, people typically use the QR-scan tool on WeChat to open any QR codes) on a sticker (the sticker is put on the wallet). Once the QR code is scanned, the browser will open a WeChat webapp where recipients can make a phone call by pressing a button on the webpage and write a text message to the owner on a text box (*SI*, Fig. S4).

To verify whether putting a WeChat link as contact information on a QR sticker is perceived to be realistic and standard in China, a preliminary study was conducted. Results showed that participants considered putting a WeChat link as contact information in a QR sticker is realistic, standard, and plausible in China. Moreover, they are significantly more likely to use WeChat than email as a contact information for their lost items (M_WeChat_ = 5.061 vs. M_email_ = 3.561; *P* < 0.00001; *SI*).

Figure 3 depicts the response rate across intervention conditions. A total of 98.3% of recipients were willing to receive the lost wallet. Thus, 11 recipients rejected to receive the wallets passed over by the research experimenters. The average QR-scan response rate was 66.3%, the average phone-call response rate was 59.3% (note that participants needed to open a WeChat page first in order to see the phone-call button), and the average message response rate was 10.9%. Among recipients who sent a text message, they also made a phone call to the owner (79.2% of messages were sent after recipients made a phone call) when their phone calls were not picked up.

Both the civic responsibility intervention (M = 65.4%) and the altruistic concern intervention (M = 61.1%) did not significantly impact recipients’ call response rate, compared to the control condition (M = 63.4%). The safety assurance intervention (M = 47.3%; χ^2^ = 8.59, *P* = 0.003) significantly decreased call response rate, compared to the control condition. We ran similar analyses on recipients’ QR-scan rates and observed similar significant effects (Fig. 3). Overall, these behavioral results are consistent with our findings in Online Experiment 2, which showed that assuring recipients of the safety of reporting a lost wallet did not lower recipients’ felt suspicion (compared to the control condition) but lowered recipients’ likelihood of reporting it. The safety assurance intervention backfired and increased the salience of safety concerns, which increased people’s reliance on it and consequently, it predominantly lowered wallet reporting behavior.

## Discussion

The current research provides evidence that researchers need to consider local norms when studying civic honesty in different cultures. Past research showed that cultural orientations are significantly correlated with lost wallet reporting behavior across countries documented in Cohn^27^. In particular, collectivism is significantly negatively correlated with reporting behavior. Recently researchers have called for not assuming that effects identified in one culture may generalize to other cultures. Building upon these findings, across one preliminary survey and three experiments, we identified local norms that are related to the lost-and-found context in China and showed that considering local norms may be important. Our data showed that the psychological mechanism underlying citizens’ honesty behavior in China may be different from what has been suggested in the literature. The content inside a lost property plays an important role in driving the decision to report it. The presence of money elevates a sense of civic responsibility and the fear of being seen as a thief if citizens fail to return it. When a lost wallet does not contain money (vs. with money), citizens are more likely to be suspicious that it is a scam. The presence of personal items elevates a sense of altruistic concern. Both civic responsibility and altruistic concern may increase reporting intention via lowering scam suspicion. When we conducted the present research, we were cautious by not assuming that any psychological mechanism for behaviors identified can be generalized across cultures. We also assume that to help citizens engage in more honesty behavior, it may be important that researchers interact with local citizens to formulate more culturally informed research questions and design^39^.

We formulate our key predictions based on preliminary study and interactions with local citizens. All three experiments were conducted in China. The primary objective of the current research is to identify effective interventions for motivating civic honesty behavior in China. Our aforementioned results were robust to excluding recipients’ characteristics and situational factors and including whether or not the experiments were run during the COVID-19 pandemic as a control (See Methods and *SI,* for robustness checks).

Our findings offer several key contributions to civic honesty literature. We provide evidence that a different psychological mechanism driving civic honesty behavior in China. Wallet content (i.e., dishonesty incentives) may shape feelings of scam suspicion and civic responsibility which play significant roles beyond the impact of altruistic concern and theft aversion. When a lost wallet does not contain money (vs. with money), citizens are more likely to be suspicious that it is a scam. This finding is provocative as it suggests that when a lost property does not contain any money but just personal items, it may evoke both altruistic concern and scam suspicion which produce opposite effects on the resultant honesty behavior. The latter could deter any empathetic citizens from reporting a lost wallet if it gets sufficiently intense. This avoidance could intensify among people with prevention orientation (e.g., those from collectivistic culture^27^). Researchers may therefore need to be cautious when interpreting behavioral patterns across different populations. Furthermore, our results suggest that the communication means between the owner and the finder may play a crucial role^33–35^. Considering the extent to which citizens from a population frequently use a means for communication; (1) WeChat app has over 1 billion monthly active users in China), (2) a local norm in China is that email is rarely used by ordinary citizens in China. Our preliminary study confirmed that Chinese participants are more likely to use WeChat than email as contact information for their lost items; they also think that using WeChat for this purpose is realistic and standard practice. Using a habitually used communication means in the intervention design could be helpful in motivating civic honesty. Thus, our findings suggest that to the extent that a communication means is habitually used, an architectural intervention such as using WeChat to report a wallet could effectively lower the friction citizens experience in their civic honesty behavioral decision, even if they suspect that the lost wallet is a scam.

Our studies examined the psychological mechanism underlying civic honesty behavior and the effects of various behavioral interventions on improving civic honesty using samples in China. Previous research showed that theft aversion and altruistic concern could drive honesty behavior. We showed that altruistic concern and theft aversion significantly mediated the impact of wallet content on participants’ tendency of engaging in civic honesty behavior. Beyond that, civic responsibility and scam suspicion additionally significantly mediated the impact of wallet content on civic honesty behavior. Based on these findings, we designed and tested the effects of three types of behavioral interventions. The altruistic concern intervention and the civic honesty intervention lowered citizens’ felt suspicion that the lost wallet is a scam. Each of these interventions indirectly increased the intention of reporting a wallet via lowering scam suspicion. The safety assurance intervention somewhat exacerbated scam suspicion concern and significantly lowered participants’ intention of reporting a lost wallet. Lastly, we conducted a field experiment to examine the effects of these behavioral interventions on civic honesty behavior. The average reporting rate is approaching 60%, suggesting that a frequently used communication means such as WeChat is an appropriate communication means for understanding and promoting civic honesty behavior in China.

The current research was the first in examining the effects of interventions on nudging civic honesty behavior. Our online experiments identified two new psychological mechanisms: wallet content (i.e., the presence of money) could lower scam suspicions and heighten felt civic responsibility. In the field experiment, we did not find a statistically positive effect of the altruistic concern intervention and the civic responsibility intervention on civic honesty behavior. We speculate that this is due to the ceiling effect of one of the interventions used. In all treatment conditions (including the control condition), we used WeChat, a communication means that citizens habitually interact with many times each day, as a choice-architectural intervention. The latter seems to be powerful in elevating civic honesty behavior. The average lost-property reporting rate across treatment conditions was 59.3%. The ceiling effect may have prevented us from examining the impact of the other behavioral interventions in the experiment. In addition, we speculate that the effectiveness of such interventions is jointly dependent on the operationalization, design, and implementation of interventions^50^. Using the current intervention design, results were not inconsistent with the existing literature. Previous field studies have shown inconsistent effects of altruistic concern interventions on prosocial behaviors (e.g., organ donations). Outside of the civic honesty context (e.g., cheating behavior in newspaper purchases and in exams), there were only a few examinations on the effects of behavioral interventions and all studies show null effects of moral reminders (such as the civic responsibility intervention used in the current research). The significant negative effect of the safety assurance intervention on civic honesty behavior is thought provoking. It did not lower suspicions of scam (Online Experiment 2) and it even lowered QR-scan and phone-call response rates of reporting the lost wallet (field experiment). The online and field experiments combine to suggest that assuring the safety of returning may heighten the salience of safety concern which is consequently used as a predominant basis for civic honesty behavior.

Our study has limitations that inform several future research avenues. First, researchers have recently argued that it is important not to assume that psychological process identified from a particular culture is generalizable to all other populations. We did not test the impact of scam suspicion and civic responsibility in other populations. Future research may investigate that. Second, our online and field experiments combine to illuminate the effects of several types of interventions: the civic responsibility and the altruistic concern interventions may increase wallet reporting intention via lowering scam suspicion, but the safety assurance intervention does not lower scam suspicion and even lower wallet reporting behavior. It is plausible that our intervention message exacerbated safety concern and therefore its effectiveness backfired. Although the negative effect is not inconsistent with our prediction, we did not form a prediction of negative effect a priori. Future research may further investigate effects of these behavioral interventions deductively. It is also plausible that the operationalization, design, and implementation of our behavioral intervention messages could be optimized to produce positive impact on the outcome behavior^50^. Third, we did not have a treatment condition in which email was used as a communication means for connecting an owner and a finder. However, our preliminary study showed that Chinese people are more likely to use WeChat than email as contact information, confirming that a local norm that email is infrequently used by ordinary Chinese citizens is relevant to the present context of interest. Our research suggests that WeChat may serve as a realistic and appropriate means for understanding and promoting civic honesty behavior in China. Future research may further explore the role of a reliable and habitually used communication means and verify whether such a choice-architectural intervention would work in other populations.

## Conclusion

Civic honesty is a crucial and diverse psychological phenomena. Our work suggests that interacting with people from a targeted culture or population is important for researchers, policymakers, and organizational leaders to formulate more culturally informed questions and design and implement more effective behavioral interventions for understanding and promoting the behavior.

## Methods

### Preliminary survey: the use of WeChat as contact information for lost items

To verify our assumption that putting a WeChat link on a QR code is a standard and preferred contact means for lost items, we conducted a survey online. Three hundred participants were recruited from an online platform, Credamo. They were shown an image of a lost wallet that we used in our experiments (a wallet that is transparent in color with a sticker; the sticker contains a QR and an intervention message). We described to participants that the sticker contained the contact information of the owner of the wallet. Upon scanning the QR, a WeChat page (called WeChat mini-program) would be opened. Thus, a finder can click a phone call button on this page to contact the owner to return the lost item. Participants indicated the extent to which the use of WeChat as a contact information (for this purpose) is realistic, plausible, and standard (all on 7-point scales). Next, we asked participants to consider if they would like to leave their contact information so that a finder could contact them when their wallet is lost, the extent to which they are likely to use such a QR-sticker (with a WeChat contact) as described. They also indicated their likelihood of using other contact means including email and phone number for their lost items. Finally, we tested whether putting a QR code on items is commonplace as perceived by Chinese citizens. Participants indicated the extent to which putting a QR code on everyday objects is common and they often see that in daily lives.

### Online experiment 1: psychological mechanism underlying civic honesty

To examine the psychological mechanism underlying civic honesty, we adapted the design in Cohn and conducted an online experiment in China in August 2019 using samples from the eight cities (Beijing, Chengdu, Guangzhou, Hangzhou, Shanghai, Shenzhen, Tianjin, and Xi’an). These were the cities where Cohn’s field experiment was conducted. We considered samples from the labor population (aged above 16) because the field experiment in the present research and related work on civic honesty are targeting the labor population. It was approved by the Institutional Review Board at Tsinghua University. The experiment was performed in accordance with relevant guidelines and regulations.

A total of 1043 participants (Mage = 29.95; female = 66.3%) who met the demographic criteria (i.e., age, gender, residence, and from the labor population) set by SoJump (wjx.cn), a large online panel that is similar to MTurk and Prolific, were randomly assigned to one of the four treatment conditions of a between-subjects design (BigMoney, NoMoney, NoKey, and Money). Thus, the wallet content was varied across four conditions as in Cohn (Table 2, for treatment conditions). Informed consent was collected before the experiment started. Participants received monetary compensation of approximately 4 USD for their participation.

**Table 2:** Treatment conditions in online experiment 1.

Conditions	Wallet content		
	Money	Amount in USD	Personal items (i.e., a key)
BigMoney	Yes	83.02	Yes
NoMoney	No	0	Yes
NoKey	Yes	13.02	No
Money	Yes	13.02	Yes

The amount of money in the Money and BigMoney conditions is based on those in Cohn et al.^6^ and had been adjusted for inflation.

The experimental design was almost identical to that in Cohn. See *SI*, Fig. S1, for how a lost wallet looks like. The most notable difference between this experiment and Cohn’s experiment in design is that instead of using a name card with the owner’s contact information, we put a sticker on which there were a QR code and a message (“Scan the QR code to contact the owner”) on the front side of each wallet. Thus, the sticker conveyed information that the wallet owner can be contacted via scanning the QR code. Note that unlike the Field Experiment in which a WeChat page would be opened once the QR code was scanned, however, participants were not to scan the QR in both Online Experiments 1 and 2 as the lost wallet scenario was imaginary.

The experimental procedures were similar to those in Cohn’s experiments. That is, participants were instructed to read a scenario that they worked in an institution (we varied the institution types) and were approached by a stranger who found a lost wallet outside the institution (the same story as in the wallet drop-off field experiment). Pictures of the wallet (with its front and its content) were displayed. After that, participants answered several blocks of questions. The same measures and scales that were used in Cohn’s experiment were administered in the current experiment. First, to ensure that participants attend to the wallet content, we asked participants to write down the items in the wallet and how much money the wallet contained (this is used as an attention check). Then, participants reported the likelihood they were to return the wallet (11-point scale, from 1 = not at all likely, to 11 = very likely; the same Likert scale was used for other 11-point measures) and the extent to which they think others would return the wallet under the same situation. The second block of questions include measures tapping into the motives underlying people’s intention of returning a lost wallet, including theft aversion, impression management, altruistic concerns, reward expectations, fear of punishment, and costs of returning the wallet identified and examined in Cohn’s experiment.

The first extension of Cohn’s experiment was the means for contacting the owner (i.e., WeChat instead of email). The consideration is that it is a local norm that email is infrequently used by Chinese citizens^33–35^. A second extension is that we considered additional motives that may underlie citizens’ motivation of returning a lost wallet. In particular, we considered scam suspicion and perceived civic responsibility. Our considerations are based upon multiple informal interviews and online searches we conducted with local people and gathered information from social media discussions (e.g., Zhihu, a Q&A online platform similar to Quora, has 109 million monthly active users) about reasons driving people’s civic honesty behaviors in China. These anecdotal evidence revealed that finders (i) tend to assess the perceived value of a lost wallet, (ii) may suspect that a lost wallet is part of a scam, and (iii) generally believe that citizens hold responsibility for protecting lost properties of others and properties of themselves. The latter two factors are consistent with well-established cultural research which showed that people from collectivistic culture tend to be more prevention focused. A prevention orientation disposes individuals to be vigilant and cultivates a sense of duty and responsibility (26–27).

In the current experiment, we measured the extent to which participants perceived the lost wallet scenario to be a scam (11-point scale). We also measured the extent to which participants were worried that the QR contained computer virus, and the extent to which they were worried that they would be accused of having pocketed money from the lost wallet. In addition, we added measures which tap into participants’ typical rule of handling lost properties—participants indicated whether it is generally considered moral to safekeep the lost property at the reception desk of the institution, they typically ignored a lost property, and they typically pretended not seeing a lost property (all with a 11-point scale). To measure participants’ perception of the owner’s and finder’s responsibility, participants reported the extent to which they thought (1) the wallet owner may come to the institution to find it, (2) it is the owner’s responsibility to find their lost wallet, and (3) it is the finder’s responsibility to return the lost wallet.

For exploratory purposes, we also measured participants’ history of losing their own wallet and that of finding a lost wallet, guess of the owner’s income level compared to the average person in their country on a seven-point scale (− 3 = much lower than the average person, + 3 = much higher than the average person), the personality trait measures of empathetic concerns and impression management^51,52^, and demographic information.

### Online experiment 2: effects of interventions and psychological mechanism

Participants were samples from China. A total of 1670 participants (Mage = 28.29; female = 50.2%) from over twenty cities (including, for example, Anhui, Beijing, Fujian, Guangxi, Guizhou, Hainan, Hebei, Henan, Heilongjiang, Hubei, Hunan, Jilin, Jiangsu, Jiangxi, Liaoning, Ningxia, Shandong, Shanxi, Shaanxi, Shanghai, Shenzhen, Sichuan, Tianjin, Zhejiang, and Chongqing) in China were recruited. They met the demographic criteria (age, gender, residence) set by SoJump, the same online platform used in Online Experiment 1. They were randomly assigned to one of eight conditions of a 2 (Wallet content: with money and a key vs. with just a key) × 4 (Interventions) between-subjects design. We recruited participants from the labor population (aged over 16) because the civic honesty field experiments in the existing literature focused on the labor population. The study was approved by the Institutional Review Board at Tsinghua University. The experiment was performed in accordance with relevant guidelines and regulations.

Informed consent was collected before the experiment started. Participants received monetary compensation of approximately USD 4 for their participation. The experiment procedures were adapted from those used in Cohn and our Online Experiment 1. One notable adaption is the means for contacting the wallet owner. As noted earlier, in Online Experiments 1 and 2, on a lost wallet there is a sticker with a QR code (in China, all QR codes can be open by a free QR-scan tool on WeChat or Alipay; Chinese citizens typically use these two apps to open QR codes) and a text message “Scan the QR code to contact the owner.” In Cohn, a business card with the owner’s email address was put inside a lost wallet and the means for contacting the wallet owner was email. Unlike the field experiment, participants were not to scan the QR code in Online Experiments 1 and 2 as the lost wallet scenario was imagery.

In this experiment, we varied two factors, (1) the amount of money present in the wallet and (2) the interventions. In the Money condition, the wallets contained RMB 93 (approximately USD 13.02), a key, and a piece of paper. In the NoMoney condition, the wallets contained just a key and a piece of paper. We developed and tested the impact of three forms of interventions: civic responsibility, altruistic concerns, and safety reassurance. There are four intervention conditions (See Maintext, Table 3, for the content of the interventions that were translated into English). They are in the form of verbal messages (in Chinese language) that are located next to the QR code in the sticker we put on each wallet. Each sticker consists of a QR code and a basic message, “Scan the QR code to contact the owner.” We add an additional message (i.e., the intervention message) on each sticker. In the Civic Responsibility Intervention conditions, we used a message, “This is a lost item. Returning a lost item is every citizen’s responsibility.” In the Altruistic Concerns Intervention conditions, we used a message, “This is a lost item. It is very precious to the owner. There is no inconsiderable loss.” In the Safety Assurance Intervention conditions, we used a message, “This is a lost item. If you are worried that this is a scam, you can call the police. Be assured that scanning the QR is safe, your money and personal information would not be stolen. Lost-and-found Centre hotline: (21) 2501 1188.” In the No Intervention (control condition), the QR code sticker only contained the basic message.

**Table 3:** Descriptions of interventions in each condition (in Chinese language).

Condition	Intervention elements
Control	Scan the QR code to contact the owner
Civic responsibility	This is a lost item. Returning a lost item is every citizen’s responsibility. Scan the QR code to contact the owner
Altruistic concern	This is a lost item. It is very precious to the owner. It can be a considerable loss to the owner. Scan the QR code to contact the owner
Safety assurance	There are two versions: (Used in Online Experiment 2) This is a lost item. If you are worried that this is a scam, you can call the police. Be assured that scanning the QR code is safe. Your money and personal information would not be stolen. Scan the QR code to contact the owner. Lost-and-Found Centre hotline: (21) 2501 1188
	(Used in Field Experiment) Scan the QR code to contact the owner of this lost item, with security assured. This Lost-and-Found WeChat Mini-program has been accredited under the Enterprise Integrity Management System

Experimental procedures were identical to those in Online Experiment 1. After reading the scenario about the lost wallet, participants answered the same questions which tapped into the psychological motives (including, theft aversion, scam suspicion, altruistic concern, civic responsibility, impression management, moral norm, fear of punishment, reward expectation, and the cost of returning) administered as in Online Experiment 1 (Individual differences in impression management and empathetic concern were not measured in this study).

### Lost wallet field experiment

A total of 660 wallets were turned in in China between September and November in 2022 (in Shanghai), and mostly between June and August in 2023 (in Beijing—Forty-seven, out of a total of 321, thus about 15% drop-offs were conducted in 2022 for the study in Beijing. The data collection was stopped because of the lock-down in Beijing in October 2022. Thus about 85% of the drop-offs in the Beijing site were conducted after the pandemic was completely over in 2023). Our study was preregistered. It was approved by the Institutional Review Board at the Chinese University of Hong Kong, Shenzhen. The experiment was performed in accordance with relevant guidelines and regulations. We selected the cities based on the criteria used in Cohn. Each of these two cities has a population of over 20,000,000, and they were also considered in Cohn. They are easy to visit and safe for our experimenters to conduct the drop-offs of wallets, and are influential politically, culturally, and economically.

#### Sample size calculation

The number of drop-off locations was determined by the population of the city, based on the formula used in Cohn (2019):

$${N}_{i}=\frac{\sqrt{{POP}_{i}}}{{\sum }_{i=1}^{j}\sqrt{{POP}_{i}}}* {N}^{target}$$

Ni is the number of drop-off locations in city *i*, POPi is the population size of city *i*, *j* is the selected city, and ${N}^{target}$ is the target sample size in a tested country. In Cohn’s main field experiments, each treatment condition has 137 to 200 drop-off locations. We therefore aimed to have 165 per cell and set the total sample size, ${N}^{target},$ to be 660 (we have four treatment conditions).

#### Selection of drop-off locations

We considered four types of societal institutions: (i) pharmacies; (ii) museums, theaters, libraries, or other cultural institutions; (iii) hotels; and (iv) public offices. We attempted to have an equal distribution of institutions. It was not entirely feasible as some areas tend to have more cultural institutions and hotels than others. In the end, we have about 10% of public offices (police stations), 29% of hotels (including three- to five-star hotels), 30% of cultural institutions (including museums, libraries, theatres), and 31% of pharmacies.

Drop-off locations and drop-off time were planned ahead. As in Cohn’s study, we used official websites, travel guide apps, and multiple popular map apps to find the relevant drop-off locations. We avoid drop-off locations that are right next to one another or located within the same block. Research experimenters travelled to different drop-off locations by subway, sharing bike, and taxi. We avoided public holidays (including weekends) as there may be traffic jams and the institutions could be busy. Before conducting the experiments, our experimenters would check whether a location still existed and planned the routes for reaching multiple drop-off locations according to the assigned drop-off time. Treatments were randomly assigned to the drop-off locations and institution types (See *SI*, Table S7, for randomization checks).

The Wallets and Drop-off Procedure.We used transparent purses as our lost wallets (see *SI*, Fig. S3). This is to ensure that the materials inside the wallet are immediately visible to recipients even if they do not open the wallet. Each wallet contained two personal items (i) two keys, and (ii) a printed sales receipt from a local convenience store. The sales receipt was intended to show that the wallet owner is a local resident. The items indicated in the sales receipt were fruits, cup noodles, bottled water, and buns.

We recruited nine experimenters to conduct the wallet drop-offs. All experimenters were undergraduate or graduate students from universities in Shanghai or Beijing. We prepared detailed experiment manuals on how to conduct the drop-offs. As in Cohn, research experimenters approached an employee of an institution and say, “Hi, I found this wallet just outside the [institution].” Experimenters then passed the wallet to the employee and said, “Somebody must have lost it. I’m in a hurry and have to go. Could you please take care of it?” Thus, instead of saying that we found the wallet inside the institution, telling recipients that we found it outside of the institution is intended to prevent recipients from thinking that the owner knew where they lost their wallet and could come to pick it up without getting notified about the location.

#### Experimental conditions

On each wallet we put a sticker which contains a QR code with a basic message, “Scan the QR code to contact the owner.” Thus, the QR code serves as a means for contacting the owner. On top of that, we added an intervention message which is located on the left side of the QR code sticker (See *SI*, Fig. S3). Based on our findings from the two online experiments, we designed four types of interventions aimed at improving civic honesty: (i) civic responsibility intervention (condition 1), (ii) altruistic concern intervention (condition 2), (iii) safety assurance intervention (condition 3), and (iv) no intervention (condition 4; See Maintext, Table 2). The civic responsibility intervention was intended to remind wallet recipients of the civic responsibility of returning lost property. The altruistic concern intervention was intended to lead recipients to imagine the feeling of losing precious personal items (e.g., highlighting that every single loss of personal items could be considerable) and thus the perceived harm recipients might bring to the owner if not returning the wallet. The safety assurance intervention was intended to assure recipients that it was safe to scan the QR to contact the owner. The control condition only contained the basic message (i.e., “Scan the QR code to contact the owner.”).

#### Measuring civic honesty

As in Cohn, the key behavioral outcome variable was whether a recipient contacted the owner to return a wallet. Our private server collected data about the interactions between recipients and the WeChat webpage, and between recipients and researchers. The QR code in each wallet had a unique ID and URL. Once scanned, a webpage on WeChat (WeChat is a popular social networking and instant messaging app since a decade ago, and has over 1 billion monthly active users) would be opened. The webpage is a form of web app (called WeChat Mini-program) run on WeChat. We developed a web app with tools such as instant messaging and phone calling.

The webpage contains information (1) about the wallet—it shows a description of the item (i.e., card case), (2) a text box which a recipient could write and send text messages to the owner, and (3) a button “call the owner” which a recipient could click to call the owner (i.e., when clicked, a recipient’s mobile phone will automatically make a phone call to the owner; *SI*, Fig. S4). Therefore, our key behavioral outcomes are whether recipients reported the wallet by scanning the QR, making phone calls, and leaving text messages on the WeChat page.

For phone calls received, we answered them by (1) thanking recipients for calling, and (2) asking recipients where they found the wallet. We also replied by telling recipients that we had already left town, the content in the wallet is not valuable, and asked the recipient to take care of it. We missed some of the phone calls and answered most of them. For text messages received, we replied to recipient’s text messages with information similar to what we told recipients in the phone calls. We recorded whether and when the QR codes were scanned, text messages, and phone calls made within 100 days after the drop-offs (Our experimenters recorded the drop-off time on a survey. Unfortunately, there were errors in the design of our survey – we provided a drop-down list of time with 30 min of increments, we should have used a text box instead to let experimenters input the exact drop-off time – and as a result we could not have accurate measures by subtracting drop-off time from phone-call time for meaningful data analyses on response time).

#### Measuring recipient characteristics and situational factors

After leaving the drop-off locations, the experimenters filled in a survey about the drop-offs. Experimenters recorded recipient gender, age (below 20, 20–30, 30–40, 40–50, over 60 years old; on a 5-point scale), the extent to which the recipient was busy (7-point scale from 0 = not at all to 6 = very busy), whether the recipient looked like a local or a foreigner (from 1 = unclear to 2 = local, to 3 = foreigner), the extent to which the recipient understood the situation (7-point scale from 0 = not at all to 6 = very clear), friendly (7-point scale from 0 = not at all to 6 = very friendly), whether the recipient was carrying a mobile phone (1 = yes, 0 = no), how many coworkers were also involved in the interaction, how many bystanders witnessed the exchange, whether a security camera was present (1 = yes, 0 = no), and whether there was a security guard (1 = yes, 0 = no) present.

## Supplementary Information

Supplementary Information.

## Data Availability

The preregistration and data analyzed in this research can be accessed via this ResearchBox link (https://researchbox.org/2291&PEER_REVIEW_passcode =WFZNBV) and will be publicly available after the manuscript is accepted for publication.
