# Publisher Correction: Randomized studies in China show that studying and promoting civic honesty needs to consider local norms

**DOI:** 10.1038/s41598-025-97127-8

**Published:** 2025-04-14

**Authors:** Iris W. Hung, Yuho Yiu, Sihan Wu, Dan Liu, Liman Wang, Xiao Han

**Affiliations:** 1 Department of Marketing, The Chinese University of Hong Kong, Shenzhen, China; 2 Department of Computer Science and Engineering, Hong Kong University of Science and Technology, Hong Kong, China; 3 Department of Marketing, Tsinghua University, Beijing, China; 4 Department of Marketing, Fudan University, Shanghai, China; 5 Hangzhou Fancha Technology, Hangzhou, China

Correction to: *Scientific Reports* 10.1038/s41598-025-87804-z, published online 29 January 2025

In the original version of this Article, the Author Contributions section was incorrect. It now reads:

“I.W.H. developed the research idea. I.W.H., Y.Y., X.H., S.W., D.L., and L.W. designed the field experiment. I.W.H., Y.Y., S.W., D.L., and L.W conducted the experiments. I.W.H., Y.Y., and L.W analyzed the data. I.W.H. and Y.Y. wrote the manuscript. These authors contributed equally: Y. Y., S. W., D. L., and L. W.”

The original Article has been corrected.

