# Supplementary material for: Randomized studies in China show that studying and promoting civic honesty needs to consider local norms
Source: Sci Rep. 2025 Jan 29;15:3710. doi: 10.1038/s41598-025-87804-z (PMC11779863; doi:10.1038/s41598-025-87804-z)
Supplement: Supplementary file 1 — Supplementary Information. [file 41598_2025_87804_MOESM1_ESM.docx]

# **Supplementary Information (SI)**

# for

Randomized studies in China show that studying and promoting civic honesty needs to consider local norms: Randomized studies in China

Supplementary Information Table of Contents

[**Preliminary Survey Results** 3](#_Toc182755534)

[**Result of Online Experiment 1** 5](#_Toc182755535)

[**Result of Online Experiment 2** 12](#_Toc182755536)

[**Results of Lost Wallet Field Experiment** 19](#_Toc182755537)

[**Survey Questions (Online Experiment 1 and 2)** 22](#_Toc182755538)

[**Survey Questions on Recipient Characteristics Filled in by Experimenters (Field Experiment)** 24](#_Toc182755539)

[**Fig. S1. How a wallet looked like in the survey of Online Experiment 1** 25](#_Toc182755540)

[**Fig. S2. How a wallet looked like in the survey of Online Experiment 2.** 26](#_Toc182755541)

[**Fig. S3. How a lost wallet and an intervention looked like (Field Experiment).** 27](#_Toc182755542)

[**Fig. S4. How the WeChat webpage looked like from the perspective of a lost-wallet finder (Field Experiment).** 28](#_Toc182755543)

[**Table S1: Treatment conditions and sample sizes in Online Experiment 2** 29](#_Toc182755544)

[**Table S2: Survey responses across experimental conditions (Online Experiment 1)** 30](#_Toc182755545)

[**Table S3: Survey responses across experimental conditions (Online Experiment 2)** 32](#_Toc182755546)

[**Table S4: Survey responses in the NoMoney conditions (Online Experiment 2)** 34](#_Toc182755547)

[**Table S5: Estimated treatment effects on civic honesty using logistic regression (Field Experiment)** 36](#_Toc182755548)

[**Table S6: Estimated treatment effects on civic honesty using linear regression (Field Experiment)** 37](#_Toc182755549)

[**Table S7: Descriptive statistics and randomization check for Field Experiment** 38](#_Toc182755550)

# **Preliminary Survey Results**

We first removed responses who failed an attention check. This leaves us with a sample of 264 participants from the original sample of 300.

***The Extent to Which the Use of WeChat as Contact Information People Put on Their Lost Items is a Standard Practice.*** We created an index on the extent to which people consider using WeChat (opened via scanning a QR) as the contact information—that people put on their items (such that if the items are lost, a finder can report them using the contact information)—is a standard practice by taking an average of the three items measured (i.e., the extent to which the use of this contact means is *realistic*, *plausible*, and *standard*, Cronbach’s α = .875). We compared this index with the midpoint of the scale (i.e., 4, using a 7-point scale: 1 = not at all, 7 = very much so) using a one-sample t-test. Results revealed that the mean index was significantly different from the midpoint (M_standard practice_ = 5.052 vs. midpoint = 4.000; t(263) = 12.94, *P* < .0001), suggesting that the use of WeChat (via scanning a QR) as contact information for lost items is considered a standard practice.

***The Likelihood of Using WeChat (Opened via Scanning a QR) as Contact Information.*** Participants indicated the likelihood that they would use such a WeChat program (via scanning a QR) as contact information for their lost items (1 = not at all likely; 7 = very likely). The mean is 5.061.

***The Likelihood of Using Email as Contact Information.*** Participants indicated the likelihood that they would use their email as contact information for their lost items (1 = not at all likely; 7 = very likely). The mean is 3.561.

***The Likelihood of Using Mobile Number as Contact Information.*** Participants indicated the likelihood that they would use their mobile number as contact information for their lost items (1 = not at all likely; 7 = very likely). The mean is 5.508.

***Which contact means are more likely to be used, WeChat, Email, or Mobile Number.*** Participants were significantly more likely to use WeChat (via scanning a QR) or their mobile phone number (M_WeChat_ = 5.061 vs. M_mobile number_ = 5.508) than their email as contact information for their lost items (M_email_ = 3.561; *P* < .00001).

***The Use of QR Codes is Commonplace in China.*** Participants indicated the extent to which they considered the use of QR codes commonplace (i.e., the extent to which they often see objects with a QR code sticker/it is common to put QR code sticker on objects). These measures were significantly greater than the midpoint of the scale (Cronbach’s α = 0.890; M = 5.019 vs. midpoint; t(263) = 10.21, *P* < .0001)

# **Result of Online Experiment 1**

We first removed responses with duplicated IP addresses. We then restricted our consideration to participants who wrote down the correct amount of money in the wallet (responses similar to the correct answers would be included). This leaves us with a sample of 960 participants from the original sample of 1,043.

***Evidence for Theft Aversion.*** Data showed evidence for theft aversion among participants. We conducted a one-way ANOVA to examine the treatment effects on the extent to which participants felt like stealing if they failed to report the lost wallet. Results largely replicated the studies conducted by Cohn et al. (*6*). Participants’ feeling of stealing if they failed to return the lost wallet significantly varies across wallet content treatment conditions (overall analysis: F(3, 956) = 9.02, *P* < 0.00001). Relative the NoMoney condition (M_NoMoney_ = 5.13, SD = 3.11), wallets carrying a large amount of money (i.e., approximately USD 83.02^[[1]](#footnote-1)^; M_BigMoney_ = 6.24, SD = 3.15) increased theft aversion (F(1, 956) = 14.46, *P* < 0.001, d = 0.35). The difference between Big Money and Money conditions (the wallet in the Money condition contained approximately USD 13.02; M_Money_ = 5.56, SD = 3.13) is also significant (F(3, 956) = 5.52, *P* = 0.019, d = 0.22). However, contrary to what is shown in Cohn et al., a wallet carrying a small amount of money (vs. no money) did not significantly increase theft aversion concern (M_money_ = 5.56, SD = 3.13 vs. M_no money_ = 5.13, SD = 3.11, *P* > .10).

Relative to the Money condition (M_Money_ = 5.56, SD = 3.13), a wallet with a small amount of money and without any key (M_NoKey_ = 6.49, SD = 3.30; F(1, 956) = 10.25, *P* = 0.001, d = 0.29) significantly increased this negative feeling of being a thief if participants failed to return a lost wallet. These results suggest that participants were more likely to feel like stealing when a wallet contained a large amount of money or when it contained just money. We do not know why the latter is the case. We speculate that participants’ theft aversion tends to depend on their perception of how likely they would be seen as a thief if not returning the wallet, and these two types of wallet content (a large amount of money or money is the only item in the wallet) were most suspicious. See *SM*, Table S2.

In sum, participants felt like stealing when a wallet contained a large amount of money or when it just contained money (i.e., no personal items).

***Evidence for Altruistic Concerns.*** Across studies, we used the extent to which participants perceived the wallet content to be precious to the owner as a proxy for altruistic concerns. Participants’ perception of how precious the wallet content is to the owner varies across wallet-content treatment conditions (F(3, 956) = 18.51, *P* < 0.00001). Relative to the NoKey condition (M_NoKey_ = 5.74, SD = 2.67), participants perceived the wallet content to be more precious to the owner when the wallet contains a key (M_Money_ = 6.56; SD = 2.33, F(1, 956) = 27.05, *P* < 0.00001, d = 0.34). This difference in altruistic concern replicates what is shown in Cohn et al. (2019).

Participants’ perception of the value of the wallet was not significantly different between the conditions in which the wallet contained money and it did not contain money (*P* > 0.10).

In sum, participants perceived the wallet to be more precious to the owner when it contained a key (i.e., personal items) versus not. Next, we tested if the data revealed evidence for two additional motives, civic responsibility, and scam suspicion.

***Evidence for Civic Responsibility.*** Data showed evidence for self-perceived responsibility for returning a lost property. An ANOVA treating participants’ felt responsibility for returning a lost property as a function of wallet-content treatment showed an overall main effect of treatment (F(3, 956) = 7.72, *P* < 0.00001). Participants reported a significantly higher level of civic responsibility of returning the wallet when the wallet contained a large amount of money (M_BigMoney_ = 7.63, SD = 1.92) than no money (M_NoMoney_ = 6.79, SD = 2.32; F(1, 956) = 18.36, *P* = 0.00002, d = 0.39). However, a wallet carrying a small amount of money (vs. no money) did not significantly increase civic responsibility (M_money_ = 6.97, SD = 2.13 vs. M_no money_ = 6.79, SD = 2.32, *P* > .10). Civic responsibility increased when a wallet contained just money (M_NoKey_ = 7.39, SD = 2.24) than when it contained both small amount of money and personal items (M_Money_ = 6.97, SD = 2.13; F(1, 956) = 4.66, *P* = 0.031, d = 0.19).

***Evidence for Scam Suspicion.*** We first conducted one-way ANOVAs treating the measure of the extent to which participants suspect the scenario to be a scam as the dependent variable and the wallet content treatment as the independent variable. Results showed that there is an overall significant main effect of treatment conditions on participants’ perception of the extent to which the wallet situation was a scam (F(3, 956) = 3.37, *P* = 0.018).

To understand the role of money present in the wallet, we compared conditions in which money is present in the wallet (vs. not). Participants were more likely to perceive the scenario to be a scam when the wallet did *not* contain money (M_NoMoney_ = 6.42, SD = 2.53) than when it contained money (compared with BigMoney: M_BigMoney_ = 5.72, SD = 2.33, F(1, 956) = 9.36, *P* = 0.002, d = 0.29; compared with Money: M_Money_ = 5.90, SD = 2.50; F(1, 956) = 5.13, *P* = 0.024, d = 0.21).

To understand the role of personal items (i.e., a key), we compared the condition in which the wallet contained a key (the Money condition) with the condition in which the wallet did not contain a key (the NoKey condition). There was no difference between these two conditions (*P* = 0.486).

In sum, results suggest that participants were significantly more likely to suspect that the lost wallet was a scam when the wallet did not contain money than when it contained money. ^[[2]](#footnote-2)^

***Stated Likelihood of Reporting a Lost Wallet.*** Participants’ stated likelihood of reporting a lost wallet appeared to be somewhat optimistic (M = 9.37, SD = 2.15; on a scale from 1 = not at all, to 11 = very likely) as in Cohn et al. Nevertheless, it could be meaningful to explore plausible motives for reporting a lost wallet. We conducted a one-way ANOVA treating participants’ reported likelihood of reporting a lost wallet as a dependent variable and the treatment as independent variables. Results revealed an overall main effect of treatment across conditions (F(3, 956) = 14.11, *P* < 0.00001). Results remain significant including or excluding covariates including (1) theft aversion, (2) altruistic concerns, (3) civic responsibility, (4) scam suspicion, (5) the extent to which participants thought they would be punished if failing to report the lost wallet, (6) the extent to which they expected they would receive a reward for reporting the lost wallet, (7) the extent to which they thought it is troublesome to report the wallet, (8) the extent to which they thought it’s the owner’s responsibility to look for their lost item, and (9) the extent to which it is moral to safekeep the lost item without contacting the owner. See *SM,* Table S2, for treatment effect estimates.

Compared to the NoMoney condition, a wallet containing a large amount of money (the BigMoney condition; M_big money_ = 9.99, SD = 1.51 vs. M_no money_ = 8.78, SD = 2.53) increased participants’ intention of reporting a lost wallet (F(1, 956) = 39.32, *P* < 0.00001, d = 0.58). Results showed similar significant effect of the presence of money when we compared the Money and the NoMoney conditions (M_money_ = 9.19, SD = 2.24; F(1, 956) = 14.11, *P* = 0.0002, d = 0.17). The presence of a key, however, did not significantly increase the wallet reporting intention (M_money_ = 9.19, SD = 2.24 vs. M_NoKey_ = 9.51, SD = 2.06; F(1, 956) = 2.79, *P* = 0.10).

Next, we attempt to understand the roles of theft aversion concerns, altruistic concerns, civic responsibility, and scam suspicion in participants’ stated likelihood of reporting a lost wallet.

***Psychological Mechanism Underlying the Stated Likelihood of Reporting a Lost Wallet.***

**The presence of personal items.** To understand the psychological process underlying participants’ stated likelihood of returning the wallet, we conducted two sets of mediation analyses. The first set examines the role of altruistic concerns, theft aversion, and civic responsibility in the impact of the presence of a key on the intention of returning the wallet. That is, we examined the indirect route of [The presence of personal items 🡪 altruistic concerns 🡪 return the lost wallet].

We restricted our observations to the Money (i.e., with money and a key) and NoKey (i.e., with just money) conditions. Mediation analyses confirmed the mediating roles of altruistic concern and civic responsibility using bootstrapped standard errors with 10,000 samples (Model 4) (*36*). Specifically, the higher the altruistic concerns, the more likely participants would return the wallet (Indirect effect of altruistic concern: β = 0.24, SE = 0.04, *P* < 0.0001, 95% CI [0.09, 0.35]). The direct effect of the presence of a key remained significant (β = -0.53, SE = 0.19, *P* = 0.0067) ^[[3]](#footnote-3)^.

**The presence of money.** The second set of mediation analyses examined the roles of scam suspicion, civic responsibility, and theft aversion in the impact of the presence of money on participants’ likelihood of returning the lost wallet. We restricted our observations to comparing the conditions with money (the BigMoney and the Money conditions combined), and the condition with no money (the NoMoney condition). We examined the indirect routes [route 1: the presence of money 🡪 the tendency of suspecting a scam 🡪 return the lost wallet], [route 2: the presence of money 🡪 thief aversion 🡪 return the lost wallet], and [route 3: the presence of money 🡪 civic responsibility 🡪 return the lost wallet]. Mediation analyses confirmed the mediating roles.

Results revealed that the *absence* of money significantly increased scam suspicion, and the higher the scam suspicion, the *less* likely participants would return the wallet (route 1: indirect effect of scam suspicion: β = -0.26, SE= 0.04, *P* < 0.0001; 95% CI [0.06, 0.28]). We also studied the role of perceived civic responsibility [the presence of money 🡪 perceived civic responsibility 🡪 return the lost wallet] and theft aversion [the presence of money 🡪 theft aversion 🡪 return the lost wallet] in the impact of the wallet content on the intention of returning the wallet. The mediating roles of civic responsibility and theft aversion were also confirmed. The presence of money significantly increased civic responsibility and theft aversion. The higher the perceived civic responsibility, the more likely participants would return the wallet (route 3: Indirect effect of civic responsibility: β = 0.26, SE = 0.04, *P* < 0.0001, 95% CI [0.05, 0.24]). The higher the theft aversion, the more likely participants return the lost wallet (route 2: Indirect effect of theft aversion: β = 0.07, SE = 0.02, *P* < 0.001; 95% CI [0.02, 0.12]). The direct effect of the presence of money remained significant (β = 0.46, SE = 0.16, *P* = 0.0052).

In sum, we identified two additional factors that could drive civic honesty behavior: scam suspicion, and civic responsibility. We replicated results of experiments in Cohn et al. by showing that altruistic concerns and theft aversion could explain people’s likelihood of returning a lost wallet. More importantly, we documented that two additional factors, scam suspicion and civic responsibility, may independently shape lost wallet returning behavior as well. Participants felt a stronger sense of civic responsibility of returning a lost wallet when the wallet contained money (vs. not), or when the wallet contained personal items (vs. not). When a lost wallet contained money (vs. not), participants were *less* suspicious that the wallet was a scam and *more* worried that they would be seen as a thief if they failed to return the wallet.

# **Result of Online Experiment 2**

Participants with duplicated IP addresses and those who did not indicate the correct amount of money (answers similar to the correct amount were included) in the wallet were excluded. This leaves us with 1,496 samples from the original one with 1,670 samples.

As in Online Experiment 1, we found evidence for civic responsibility, altruistic concerns, scam suspicion, and theft aversion, and showed mediating effects of these factors. In particular, altruistic concerns, civic responsibility, and theft aversion independently mediates the impact of the presence of personal items (i.e., a key) on participants’ likelihood of returning the wallet, whereas civic responsibility, theft aversion, and scam suspicion independently mediates the impact of the presence of money (especially large amount of money) on the likelihood of returning the wallet. See *SM,* Table S3 and S4, for treatment effect estimates.

***Evidence for theft aversion.***  A 2 × 4 ANOVA on participant’s feeling of stealing the lost wallet revealed a significant main effect of the presence of money (F(1, 1488) = 30.83, *P* < 0.00001, d = 0.29). Participants have a stronger feeling of being a thief if they failed to return the lost wallet when the wallet contained money (M_Money_ = 5.10, SD = 3.30) than when it did not (M_NoMoney_ = 4.16, SD = 3.18). The main effect of interventions and the interaction were not significant (*P*s > 0.90).

***Evidence for altruistic concerns.*** We conducted a 2 × 4 ANOVA treating participant’s perception that the wallet is precious to the owner as a dependent variable, and the presence of money contained in the wallet and interventions as independent variables. The presence of money had no significant effects on the perceived value of the lost wallet to the owner in the eyes of participants. Both the main effect of the presence of money (F(1, 1488) = 3.36, *P* = 0.067) and its interaction (F(3, 1488) = 1.29, *P* = 0.275) with interventions did not reach significance. These results are consistent with our predictions as wallets in both money conditions contained the same personal items (i.e., all wallets contained a key).

More importantly, results revealed a significant main effect of intervention (F(3, 1488) = 2.75, *P* = 0.041). The altruistic concern intervention significantly increased the perceived value of the lost wallet to the owner (M_altruistic_ = 6.34, SD = 2.24) in the eyes of participants, compared to the civic responsibility intervention condition (M_civic responsibility_ = 5.98, SD = 2.43), the safety assurance intervention condition (M_safety assurance_ = 5.89, SD = 2.32), or the control condition (M_control_ = 5.97, SD = 2.35; Fs(1, 1488) > 4.54, *P* < 0.033, d > 0.16 for all pairwise comparisons).

***Evidence for civic responsibility.*** We conducted a 2 × 4 ANOVA treating participant’s perceived civic responsibility of returning a lost item as the dependent variable, and the presence of money contained in the wallet and interventions as independent variables. Results revealed a significant main effect of the presence of money (F(1, 1488) = 9.02, *P* = 0.003). Participants perceived a stronger sense of civic responsibility when the wallet contained money than when it did not (M_Money_ = 6.14, SD = 2.31 vs. M_NoMoney_ = 5.78, SD = 2.28, F(1, 1488) = 9.02, *P* = 0.003, d = 0.16). The main effect of interventions and the 2-way interaction were not significant (*P*s > 0.444).

***Evidence for scam suspicion.*** Participants indicated the extent to which they suspected that it was a scam. A 2 × 4 ANOVA on scam suspicion revealed a significant main effect of the presence of money (F(1, 1488) = 29.43, *P* < 0.00001, d = 0.28), and a significant main effect of interventions (F(3, 1488) = 4.97, *P* = .002). The two-way interaction was not significant (*P* = 0.262). Participants were more likely to suspect that the lost wallet scenario was a scam when the wallet did not contain money than when it contained money (M_NoMoney_ = 5.81, SD = 2.42 vs. M_Money_ = 5.12, SD = 2.50).

Moreover, the interventions shaped participants’ perception of scam suspicion. When the civic responsibility intervention (M_civic responsibility_ = 5.16, SD = 2.46) was used, participants felt a lower suspicion of scam than when the control message (i.e., no intervention message; M_control_ = 5.64, SD = 2.54; F(1, 1488) = 7.28; *P* = 0.007, d = 0.19), or the safety assurance intervention (M_safety assurance_ = 5.76, SD = 2.44; F(1, 1488) = 11.40, *P* < 0.001, d = 0.24) was used. When the altruistic concern intervention (M_altruistic_ = 5.30, SD = 2.44) was used, participants felt a lower suspicion of scam than when the control message (M_control_ = 5.64, SD = 2.54; F(1, 1488) = 3.54, *P* = 0.059, d = 0.14) or the safety assurance intervention (M_safety assurance_ = 5.76, SD = 2.44; F(1, 1488) = 6.56, *P* = 0.01, d = 0.19) was used. Safety assurance intervention did not lessen any scam suspicion significantly (M_safety assurance_ = 5.76, SD = 2.44 vs. M_control_ = 5.64, SD = 2.54; F < 1, *P* = 0.49). See *SM*, Table S3 and S4, for treatment effect estimates.

In sum, compared to the control condition, both the civic responsibility intervention and the altruistic concern intervention decreased felt suspicion of scam, whereas the safety assurance intervention did not decrease scam suspicion. We do not think the latter is contrary to our expectation as the safety assurance intervention may have actually increased safety concerns and thus people’s reliance on scam suspicion on their wallet reporting decision. To understand the nature of the effects of the interventions, we will come back to that in the *Psychological Mechanism* section next.

***Stated intention for reporting a lost wallet.*** We conducted a 2 × 4 ANOVA treating participant’s intention of returning a lost item as the dependent variable, and the presence of money contained in the wallet and interventions as independent variables. Results revealed a significant main effect of the presence of money (F (1, 1488) = 48.10, *P* < 0.0001, d = 0.35) and a significant main effect of interventions (F (3, 1488) = 17.56, *P* < 0.0001). Participants were more likely to return the wallet when it contained money (M_Money_ = 8.36, SD = 2.26) than when it did not contain any money (M_NoMoney_ = 7.50, SD = 2.59, d = 0.35). The two-way interaction was not significant (*P* = 0.264).

Participants were less likely to return the wallet when the safety assurance intervention was used. The safety assurance intervention significantly decreased participants’ likelihood of returning the wallet (M_safety assurance_ = 7.13, SD = 2.74) compared to the control condition (M_control_ = 8.17, SD = 2.40; F(1, 1488) = 34.56, *P* < .0001, d = 0.40).

Beliefs in others’ likelihood of returning the wallet showed a similar pattern of results. The main effects of the presence of money and the main effect of interventions were significant (F(1, 1488) = 9.19, *P* = 0.002 and F(3, 1488) = 8.77, *P* < 0.00001 respectively). The two-way interaction was not significant (*P* > 0.70). Participants believed that others were more likely to return the wallet when it contained money (M_Money_ = 6.85, SD = 2.19) than when it did not contain any money (M_NoMoney_ = 6.49, SD = 2.31, d = 0.16). Participants’ belief that others will be returning the wallet decreased when the safety assurance intervention was used. The safety assurance intervention significantly decreased participants’ belief of others’ intention of returning the wallet (M_safety assurance_ = 6.16, SD = 2.39), compared to the civic responsibility intervention condition (M_civic responsibility_ = 6.88, SD = 2.36), the altruistic concerns intervention condition (M_altruistic_ = 6.72, SD = 2.16), or the control condition (M_control_ = 6.91, SD = 2.13).

***Psychological Mechanism.*** To examine the psychological mechanism, we conducted mediation analyses to examine the psychological mechanisms underlying the effects of the interventions on participants’ intention of returning the wallet.

**Effects of civic responsibility intervention.** As noted earlier, participants showed lower scam suspicion when the civic responsibility intervention (vs. control) was used. We restricted our observations to the civic responsibility intervention conditions and the control conditions. The indirect effects of civic responsibility with routes [civic responsibility intervention 🡪 scam suspicion 🡪 return the wallet] was examined. A mediation analysis confirmed the mediating role of scam suspicion and the nature of the effects of civic responsibility intervention using bootstrapped standard errors with 10,000 samples (Model 4) (*28*). The civic responsibility intervention lowered scam suspicion, and consequently increases the likelihood of returning (indirect effect route: β = -0.27, SE = 0.04, 95% CI [0.04, 0.25]). The direct effect of the civic responsibility intervention became non-significant (*P* = 0.679). In sum, the civic responsibility intervention lowers scam suspicion and consequently could lift the intention of returning the wallet.

**Effects of the altruistic concerns intervention.** As noted earlier, participants perceived the lost wallet to be more precious and showed *less* scam suspicion when the altruistic concerns intervention was used (vs. no intervention). Here, we restricted our observations to the altruistic concerns intervention conditions and the control conditions. The indirect effects of altruistic concern with routes [route 1: altruistic concerns intervention 🡪 perceived harm to the owner 🡪 return the wallet] and [route 2: altruistic concerns intervention 🡪 perceived harm to the owner 🡪 scam suspicion 🡪 return the wallet] were considered. We conducted serial mediation analyses to examine the roles of altruistic concerns and scam suspicion. Their mediating roles were confirmed (indirect route 1: [altruistic concerns intervention 🡪 perceived harm to the owner 🡪 return the wallet], Effect = 0.09, Boot SE = 0.04, a 95% CI [0.01; 0.19]; indirect route 2 [altruistic concerns intervention 🡪 perceived harm to the owner 🡪 scam suspicion 🡪 return the wallet], Effect = 0.02, Boot SE = 0.01, 95% CI [0.004, 0.05]). The direct effect of the altruistic concern intervention became non-significant (*P* = 0.222). In sum, the altruistic concern intervention lowered scam suspicion and consequently could lift the likelihood of returning the wallet.

**Effects of the safety assurance intervention.** Participants’ likelihood of returning the lost wallet decreases when the safety assurance intervention (vs. control) was used (M_safety assurance_ = 7.13, SD = 2.74 vs. M_control_ = 8.17, SD = 2.40; F(1, 1488) = 34.56, *P* < 0.0001, d = 0.40). In addition, this intervention did not significantly lower scam suspicion (M_safety assurance_ = 5.76, SD = 2.44 vs. M_control_ = 5.64, SD = 2.54). We expected that assuring participants about the safety of returning the lost wallet would lessen feelings of suspicion and therefore motivate them to return the lost wallet. It is plausible that assuring participants about the safety for reporting a lost wallet actually backfired. The intervention may have increased the salience of safety concerns which exacerbated participants’ reliance on it as a basis for their civic honesty behavior. Data supported the validity of the latter assumption. The wallet reporting intention (as dependent variable), the presence of safety assurance intervention, participants’ perceived suspicion, and their interaction terms (as independent variables) were considered in a moderation analysis (Model 1) (*28*). Results supported our prediction: the presence of a safety assurance intervention (vs. control) significantly enhances participants’ reported likelihood of reporting a lost wallet. The presence of safety assurance intervention significantly moderated the impact of perceived scam suspicion on reporting intention (interaction: β = -0.20, SE = 0.08, *P* = 0.013). In particular, the presence of safety assurance intervention (vs. control) significantly enhanced the negative impact of perceived scam suspicion on wallet reporting intention (β = -0.496, SE = 0.059, *P* = 0.0000, a 95% CI [-0.61, -0.38]; see *Maintext,* Fig. 2 for path analyses).

To summarize, we obtained evidence about the effects of the interventions on participants’ stated likelihood of returning the lost wallet. Reminding participants of civic responsibility or altruistic concerns could effectively lower scam suspicion and consequently lift their intention of returning it. Assuring participants of the safety of reporting a lost wallet, however, could increase alertness to safety concerns and consequently decreased their intention of returning it.

# **Results of Lost Wallet Field Experiment**

We intended to drop off 660 wallets. Two wallets were not assigned to a drop-off location due to handling mistakes and were excluded. This led to a total of 658 samples remaining.

***Response rates of reporting a lost wallet.*** To report a wallet, recipients needed to first scan the QR code. After that, their WeChat would open a webpage (with text messaging and phone-call functions) where recipients could send a text message and call the owner (*SM,* Fig. S4).

On average, 66.3% of recipients scanned the QR code, 10.9% of recipients sent a text message to the owner, and 59.3% of recipients made a call to the owner to report a wallet. Recipients’ responses rates of *calling the owner* were analyzed as a function of intervention conditions in a binary logistic regression. Results revealed a main effect of intervention (χ²(3) = 13.60, *P* = 0.004). Response rates via making a call to the owner in the altruistic concern intervention (61.1% of recipients) and in the civic responsibility (65.4 % of recipients) were not significantly different from that in the control condition (63.4% of recipients). However, response rate significantly decreased when safety assurance intervention (47.3%; χ²(1) = 8.59, *P* = 0.003) was used, compared to the control condition. There was no significant difference in recipients’ rate of *sending a text message* among the four intervention conditions (*P* = 0.251). For those who sent a text message, they all made a phone call to the owner. Our research assistants answered most of the calls but missed some of them. We observed that when a call was missed, recipients followed up by writing a text message to the owner.

Recipients’ response rates of *scanning the QR* was analyzed as a function of intervention conditions in a binary logistic regression. Results revealed a main effect of intervention (χ²(3) = 14.96, *P* = .002). There was no significant difference among the civic responsibility intervention condition (71.6%), the altruistic concern intervention condition (68.9%), and the control condition (70.7%). The safety assurance intervention lowered recipients’ likelihood of scanning the QR code (54%), compared to the control condition (χ²(1) = 9.75, *P* = 0.002). See *SM*, Table S5 and S6.

***Robustness checks.*** We conducted robustness checks to see if the results observed above remain after controlling for recipient characteristics and situational factors that the experimenters recorded. A binary logistic regression treating recipients’ rate of calling the owner as the dependent variable (yes = 1; no = 0), intervention as the independent variable, and control variables including recipient gender, age, busyness, the extent to which they understood the situation, the extent to which recipients were friendly, the extent to which they looked suspicious, whether recipients were carrying mobile phones, the number of coworkers handling the wallet together, the number of observers witnessing the drop-offs, whether there was a security camera, and whether there was a security guard nearby. The same effects of our behavioral intervention were observed even after controlling for these control variables. That is, compared to the control conditions, the civic responsibility intervention and the altruistic concern intervention did not significantly increase call response rates (*P*s > 0.210), and the safety assurance intervention remained to significantly decreased call response rates (χ² (1) = 7.34; *P* = 0.007). As discussed in the main text, highlighting the safety of reporting a wallet seems to backfire by increasing the salience of safety concerns which consequently decreased recipients’ call response rates. Recipients’ age significantly decreased call response rates (χ² (1) = 8.44, *P* = 0.004). The older the recipients were, the less likely they were to report the wallet. No other effects of these recipients’ characteristics and situational factors were significant (see *SM*, Table S5 and S6).

In sum, effects of interventions were robust after including or excluding recipients’ characteristics and situational factors.

# **Survey Questions (Online Experiment 1 and 2)**

Survey questions (in Chinese language) of Online Experiments 1 and 2 are shown in the following order:

1. Please take a look at the picture shown and answer the following questions:
   1. Please write down the content of the wallet in the space below
   2. Please write down the amount of money in the wallet (if no money, please indicate 0)?
2. How likely is it that you contact the owner to return the lost property? [0 = not at all; 10 = very likely]
3. How likely is it that others would contact the owner to return the lost property under a similar situation? [0 = not at all; 10 = very likely]
4. To what extent do you think that the lost wallet scenario is a scam? [0 = not at all; 10 = very likely]
5. To what extent are you worried that the owner would accuse you of having pocketed the money in the wallet? [0 = not at all; 10 = very likely]
6. To what extent are you worried that the QR link contains computer virus? [0 = not at all; 10 = very likely]
7. To what extent do you think it is moral to just safekeep the lost item at the reception? [0 = not at all moral; 10 = very moral]
8. To what extent do you think you should bypass and pretend not seeing the lost item? [0 = not at all; 10 = very much]
9. To what extent do you think it is moral to avoid a lost wallet (i.e., pretend not seeing it and do not contact the owner)? [0 = not at all moral; 10 = very moral]
10. To what extent do you think that owners will come find it even if no one contacts them? [0 = not at all; 10 = very much]
11. To what extent do you think that it is the owner's responsibility of finding the lost item? [0 = not at all; 10 = very much]
12. To what extent do you think that it is your responsibility to contact the owner to return the lost item? [1 = not at all; 11 = very much]
13. To what extent do you think that you are likely to receive monetary reward from the owner if returning the lost item? [1 = not at all; 11 = very much]
14. If you do not contact the owner, to what extent are you worried about others' impression on you? [1 = not at all worried, 11 = very worried]
15. To what extent do you feel like stealing if you fail to return the lost wallet? [1 = not at all, 11 = very much]
16. To what extent do you think that the wallet is important to the owner? [1 = not at all important, 11 = very important]
17. To what extent do you think that the wallet is precious to the owner? [1 = not at all precious, 11 = very precious]
18. To what extent do you worry that you will be punished if you do not contact the owner? [1 = not at all worried, 11 = very worried]
19. To what extent do you think that it is troublesome to return the lost wallet? [1 = not at all troublesome, 11 = very troublesome]
20. Please indicate whether the wallet contains the following items:
    1. Key [yes/no]
    2. QR code [yes/no]
    3. Money [yes/no]
    4. Paper [yes/no]
21. Have you lost a wallet, mobile phone, or keys before? [yes/no]
22. Have you ever found a lost wallet, mobile phone, or keys before? [yes/no]
23. Please estimate the income level of the owner compared to the national average income level [-3 = far lower than the average, 3 = far more than the average]
24. Empathetic concern subscale from the Interpersonal Reactivity Index (items 1 to 7) [1 = disagree, 5 = agree very much] (ref 3)
25. Impression management subscale of the Balanced Inventory of Desirable Responding (items 1 to 10) [1 = not at all correct, 7 = very correct] (ref 4)
26. Your age (please write down in the space below)
27. Your gender [male/female]
28. Ethnic group (please write down in the space below)
29. What is the highest degree you are pursuing/you already obtained? [below senior high school/ graduated from senior high school/ college level/ university undergraduate level/ university master level/ university doctoral level]
30. What is the status of your employment? [student, unemployed/ finding a job, internship/ employed/ self-employed/ freelance/ out of employment (due to health issue)/ out of employment (retired)/ out of employment, reason___]
31. City (please write down in the space below)
32. Household income level (before tax): [less than 50,000 rmb/ 50000 to 100000 rmb/ 100000 to 200000 rmb/ 200000 to 400000 rmb/ 400000 to 800000 rmb/ more than 800000 rmb]

Note: Questions in point #24 and #25 were not administered in Online Experiment 2; the 11-point scales were from 0 to 10 in Online Experiment 2.

# **Survey Questions on Recipient Characteristics Filled in by Experimenters (Field Experiment)**

Survey questions (in Chinese language) filled in by experimenters after drop-offs:

1. Research assistant ID
2. Site (Shanghai/Beijing)
3. Please indicate the wallet drop off time
4. Recipient’s gender (male/female)
5. Age: [less than 20/ 20 - 30 yrs old/ 30 to 40 yrs old/ 40 - 50 yrs old/ 50 - 60 yrs old/ more than 60 yrs old]
6. To what extent was the recipient busy? (0 = not at all, 6 = very busy)
7. To what extent do you think that the recipient is a local resident? (not sure, or not local/ yes)
8. To what extent was the recipient clear about the RA’s description of the lost wallet scenario? (0 = not at all, 6 = very clear)
9. To what extent the recipient was friendly? (0 = not at all friendly, 6 = very friendly)
10. To what extent the recipient was suspicious? (0 = not at all, 6 = very suspicious)
11. Did the recipient carry a mobile phone? (yes/no)
12. Were there coworkers involved in the communication? (none/ 1/ 2/ 3/ more than 3)
13. How many observers who witnessed the interaction? (none/ less than 5/ more than 5)
14. Were there security camera? (yes/no)
15. Were there any security guard? (yes/no)

**
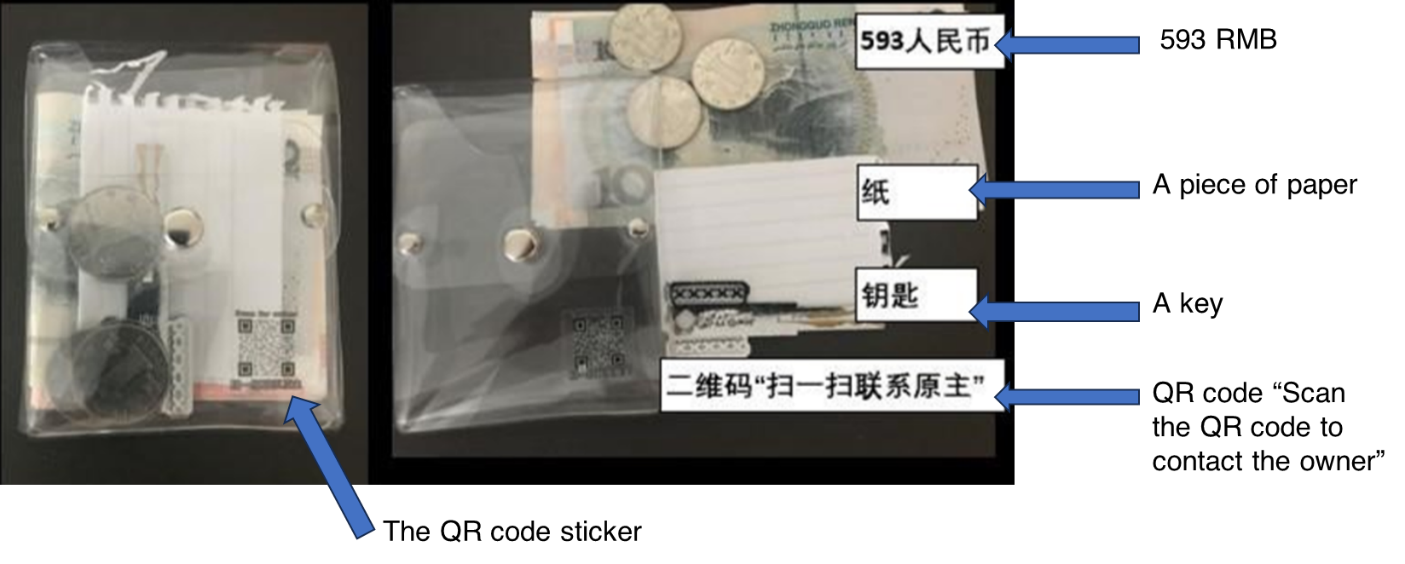
**

**Fig. S1. How a wallet looked like in the survey of Online Experiment 1.** This is shown in the BigMoney condition. Note that the lost wallet scenario was imaginary in Online Experiment 1 and therefore participants did not have to scan any QR code.


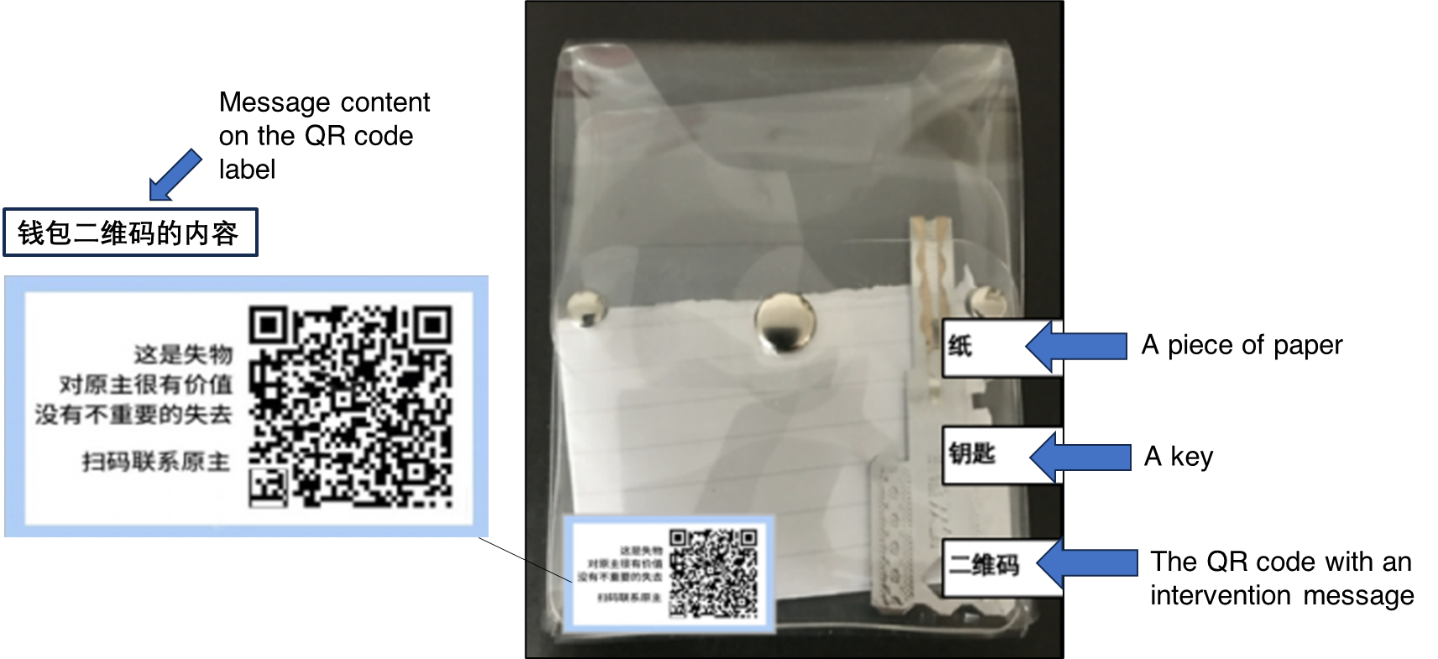


**Fig. S2. How a wallet looked like in the survey of Online Experiment 2.** This is shown in the NoMoney-AltruisticConcern condition. Note that the lost wallet scenario was imaginary in Online Experiment 2 and therefore participants did not have to scan any QR code. The specific intervention message (altruistic concern intervention in this figure) was also enlarged and displayed on the right-hand side the lost wallet image.


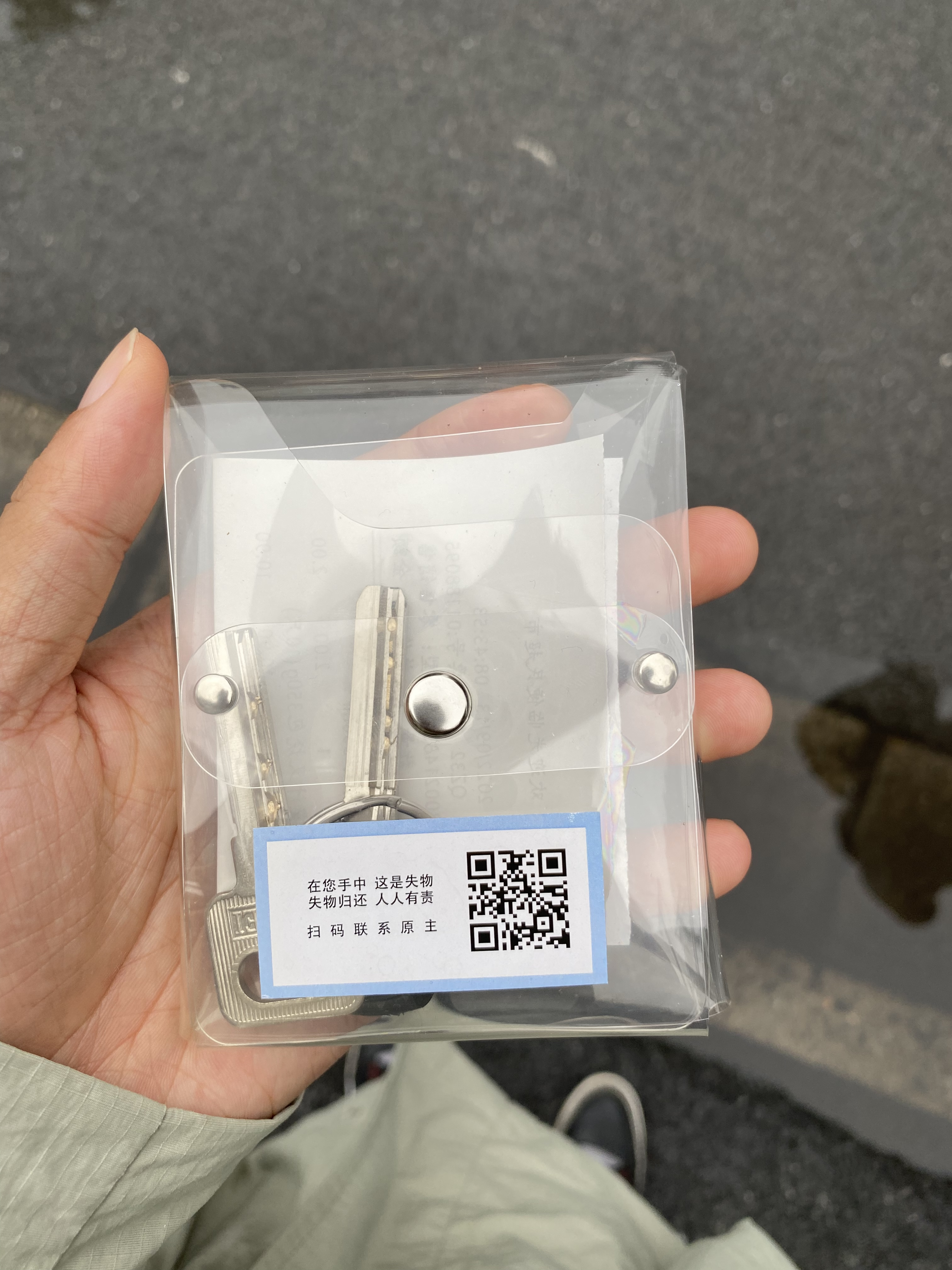


**Fig. S3. How a lost wallet and an intervention looked like (Field Experiment).** A lost wallet is a plastic card case. Its content includes two keys and one printed sales receipt from a convenience store. The intervention shown in the figure is the civic honesty intervention.

**
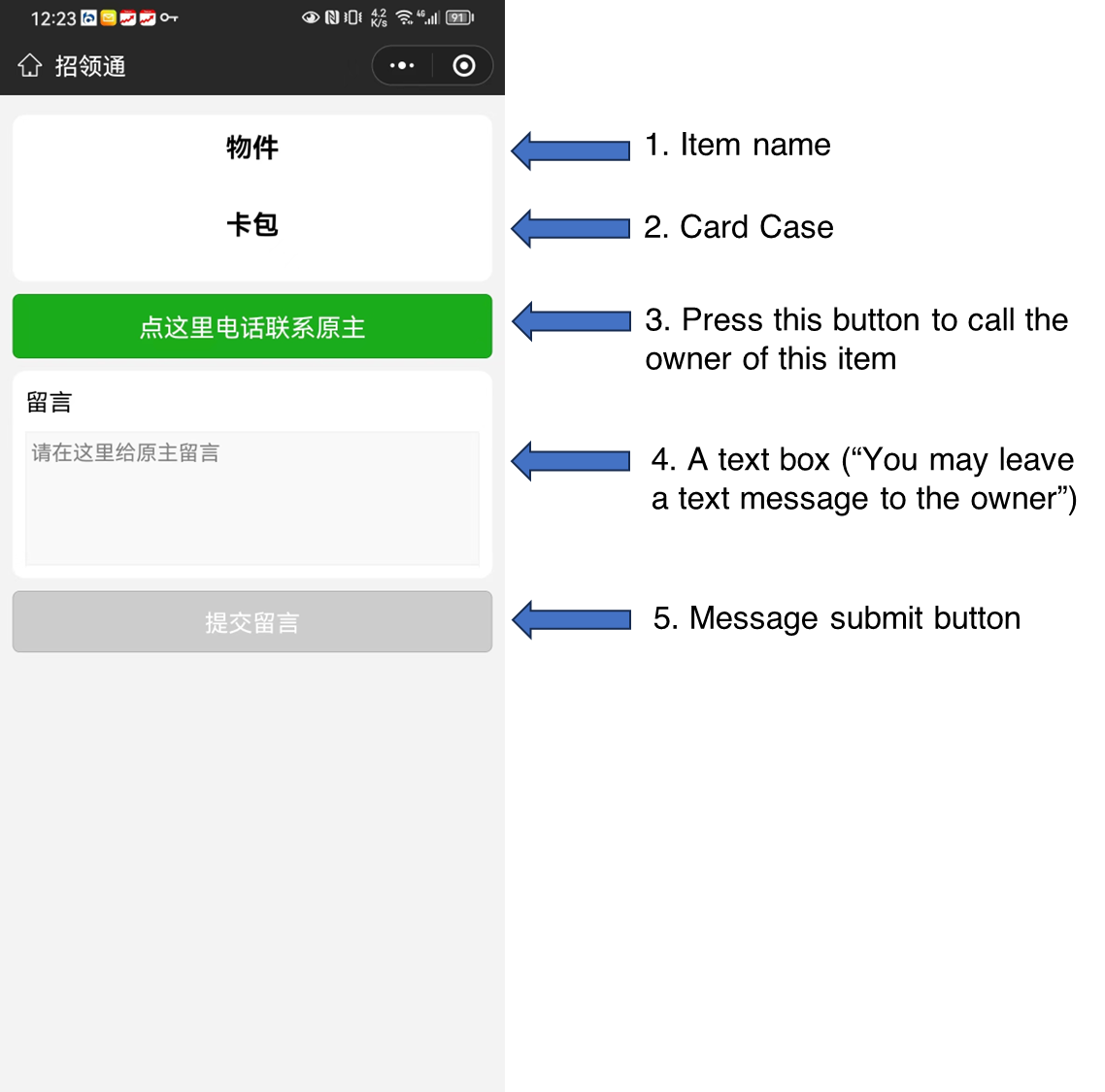
**

**Fig. S4. How the WeChat webpage looked like from the perspective of a lost-wallet finder (Field Experiment).** Once the QR (on a lost wallet) is scanned, a webpage (a form of web-application, run on WeChat without installations of App) will be opened. (**1**) a label: item name, (**2**) we set “card case” to be the item name for all wallets, (**3**) a phone call will be automatically made (thus the owner did not need to type any phone number) to call the owner, (**4**) a text box where the finder can write to the owner, (**5**) the message submission button.

#

# **Table S1: Treatment conditions and sample sizes in Online Experiment 2**

| **Intervention Conditions** | **Wallet Content** | | | |  |
| --- | --- | --- | --- | --- | --- |
|  | | Money and Key | | Key Only | |
| Civic Responsibility | | 195 | 189 | | |
| Altruistic Concern | | 186 | 186 | | |
| Safety Assurance | | 181 | 183 | | |
| Control | | 192 | 184 | | |

#

# **Table S2: Survey responses across experimental conditions (Online Experiment 1)**

|  | Altruistic concerns | Altruistic concerns | Civic responsibility | | Scam  suspicion | Theft  aversion | Stated likelihood of reporting | | | |
| --- | --- | --- | --- | --- | --- | --- | --- | --- | --- | --- |
|  | (1) | (2) | (3) | | (4) | (5) | (6) | (7) | (8) | (9) |
| Money | 0.862****  (0.234) | -0.266  (0.213) |  | 0.184  (0.204) | -0.537** (0.231) | 0.440  (0.286) | -0.528***  (0.193) | 0.412*  (0.219) | 0.260  (0.188) | 0.289  (0.189) |
| BigMoney |  | 0.477**  (0.207) | ** | 0.844***** (0.197) | -0.696 (0.223)*** | 1.103***** (0.288) |  | 1.210***** (0.192) | 0.741***** (0.167) | 0.824*****  (0.167) |
| Money-NoKey |  | -1.115*****  (0.233) | ***** | 0.597*** (0.213) | -0.328 (0.243) | 1.350***** (0.300) |  | 0.718**** (0.215) | 0.581***  (0.190) | 0.582***  (0.190) |
| Theft aversion |  |  |  | |  |  |  |  | 0.067****  (0.021) | 0.082****  (0.022) |
| Altruistic concerns |  |  |  | |  |  | 0.245*****  (0.044) |  | 0.135*****  (0.030) | 0.137*****  (0.030) |
| Civic responsibility |  |  |  | |  |  |  |  | 0.207*****  (0.036) | 0.200*****  (0.036) |
| Scam suspicion |  |  |  | |  |  |  |  | -0.223*****  (0.029) | -0.205*****  (0.030) |
| Fear of punishment |  |  |  | |  |  |  |  |  | -0.010  (0.025) |
| Reward expectation |  |  |  | |  |  |  |  |  | -0.063***  (0.024) |
| Owner’s responsibility |  |  |  | |  |  |  |  |  | 0.041  (0.028) |
| Cost of reporting |  |  |  | |  |  |  |  |  | -0.070**  (0.028) |
| Moral to safekeep w/o reporting |  |  |  | |  |  |  |  |  | 0.081**  (0.026) |
| Constant | 5.382*****  (0.350) | 6.609*****  (0.234) | 6.700*****  (0.221) | | 6.810*****  (0.267) | 4.998*****  (0.314) | 8.147*****  (0.401) | 8.650***** (0.239) | 7.550***** (0.403) | 7.264*****  (0.505) |
| Control:  Institution FE | Yes | Yes | Yes | | Yes | Yes | Yes | Yes | Yes | Yes |
| Observations | 483 | 960 | 960 | | 960 | 960 | 483 | 960 | 960 | 960 |
| Adjusted R^2^ | 0.025 | 0.055 | 0.020 | | 0.010 | 0.024 | 0.080 | 0.039 | 0.262 | 0.279 |

*Notes:* OLS estimates with robust standard errors in parentheses. The omitted category for column (1) and (6) is the treatment “**NoKey**” (i.e., with just money). The omitted category for column (2) to (5), and (7) to (9) is the treatment “**NoMoney**” (i.e., with just a key). Control variables include fear of punishment, reward expectation, owner’s responsibility, cost of reporting a wallet, and the perception that safekeeping a wallet without reporting it is moral. Institutions fixed effect was also included. Significance level: ***** *P* < 0.0001; **** *P* < 0.001, *** *P* < 0.01, ** *P* < 0.05, * *P* < 0.10.

# **Table S3: Survey responses across experimental conditions (Online Experiment 2)**

|  | Altruistic concerns | Civic responsibility | Scam  suspicion | Theft  aversion | Stated likelihood of reporting | | | | |
| --- | --- | --- | --- | --- | --- | --- | --- | --- | --- |
|  | (1) | (2) | (3) | (4) | (5) | | (6) | | (7) |
| Money | -0.270  (0.243) | 0.499  (0.240) | -0.804***  (0.260) | 1.024**  (0.341) | | 1.031*****  (0.244) | | 0.702***  (0.214) | 0.701****  (0.213) |
| Altruistic concern intervention | 0.510**  (0.227) | 0.111  (0.238) | -0.544**  (0.251) | -0.034  (0.330) | | 0.257  (0.252) | | 0.010  (0.211) | 0.030  (0.210) |
| Civic responsibility intervention | -0.029  (0.248) | 0.380*  (0.230) | -0.642**  (0.254) | 0.022  (0.330) | | 0.221  (0.258) | | -0.059  (0.224) | -0.031  (0.225) |
| Safety assurance intervention | -0.278  (0.249) | 0.025  (0.245) | 0.235  (0.254) | -0.010  (0.339) | | -1.079****  (0.282) | | -0.987  (0.235) | -1.017*****  (0.235) |
| Altruistic concerns |  |  |  |  |  | | | 0.145*****  (0.028) | 0.133*****  (0.029) |
| Civic responsibility |  |  |  |  |  | | | 0.323*****  (0.032) | 0.317*****  (0.032) |
| Scam suspicion |  |  |  |  |  | | | -0.253*****  (0.026) | -0.245*****  (0.026) |
| Theft aversion |  |  |  |  |  | | | 0.003  (0.019) | 0.002  (0.022) |
| Fear of punishment |  |  |  |  |  | |  | | 0.026  (0.024) |
| Reward expectation |  |  |  |  |  | |  | | -0.007  (0.025) |
| Owner’s responsibility |  |  |  |  |  | |  | | 0.058**  (0.028) |
| Cost of reporting |  |  |  |  |  | |  | | -0.055**  (0.023) |
| Moral to safekeep w/o reporting |  |  |  |  |  | |  | | 0.054**  (0.025) |
| Constant | 6.109*****  (0.171) | 5.652*****  (0.168) | 6.049*****  (0.183) | 4.169*****  (0.237) | | 7.641*****  (0.185) | | 6.448*****  (0.314) | 6.000*****  (0.438) |
| Observations | 1496 | 1496 | 1496 | 1496 | 1496 | | 1496 | | 1496 |
| Adjusted R^2^ | 0.006 | 0.004 | 0.003 | 0.016 | 0.062 | | 0.601 | | 0.304 |

*Notes:* OLS estimates with robust standard errors in parentheses. The omitted category is the treatment “**NoMoney**” (i.e., with just a key) for the presence of money variable and control intervention (i.e., no intervention) for the intervention variable. Interaction terms were included but not shown for ease of exposition. Control variables include fear of punishment, reward expectation, owner’s responsibility, cost of reporting a wallet, and the perception that safekeeping a wallet without reporting it is moral. Institutions fixed effect was also included. The interaction terms involving Money and Intervention conditions were included in all models. Significance level: ***** *P* < 0.0001; **** *P* < 0.001, *** *P* < 0.01, ** *P* < 0.05, * *P* < 0.10.

# **Table S4: Survey responses in the NoMoney conditions (Online Experiment 2)**

|  | Altruistic concerns | Civic responsibility | Scam  suspicion | Theft  aversion | Stated likelihood of reporting | | | | | |
| --- | --- | --- | --- | --- | --- | --- | --- | --- | --- | --- |
|  | (1) | (2) | (3) | (4) | (5) | | (6) | | (7) | |
| Altruistic concern intervention | 0.510**  (0.227) | 0.111  (0.238) | -0.544**  (0.251) | -0.034  (0.330) | | 0.257  (0.252) | | -0.027  (0.212) | | -0.022  (0.212) |
| Civic responsibility intervention | -0.029  (0.248) | 0.380*  (0.230) | -0.642**  (0.254) | 0.022  (0.330) | | 0.221  (0.258) | | -0.079  (0.226) | | -0.094  (0.229) |
| Safety assurance intervention | -0.278  (0.249) | 0.025  (0.245) | 0.235  (0.254) | -0.010  (0.339) | | -1.079****  (0.282) | | -0.973*****  (0.235) | | -1.011*****  (0.236) |
| Altruistic concerns |  |  |  |  | |  | | 0.203*****  (0.044) | | 0.193*****  (0.044) |
| Civic responsibility |  |  |  |  | |  | | 0.384*****  (0.048) | | 0.362*****  (0.049) |
| Scam suspicion |  |  |  |  | |  | | -0.251*****  (0.039) | | -0.238*****  (0.040) |
| Theft aversion |  |  |  |  | |  | | -0.022  (0.029) | | -0.028  (0.033) |
| Fear of punishment |  |  |  |  | |  | |  | | 0.024  (0.037) |
| Reward expectation |  |  |  |  | |  | |  | | 0.059*  (0.035) |
| Owner’s responsibility |  |  |  |  | |  | |  | | 0.061  (0.042) |
| Cost of reporting |  |  |  |  | |  | |  | | -0.090***  (0.034) |
| Moral to safekeep w/o reporting |  |  |  |  | |  | |  | | 0.008  (0.036) |
| Constant | 6.109*****  (0.171) | 5.652*****  (0.168) | 6.049*****  (0.183) | 4.169*****  (0.237) | | 7.641*****  (0.185) | | 5.841*****  (0.441) | | 5.706*****  (0.590) |
| Observations | 742 | 742 | 742 | 742 | | 742 | | 742 | | 742 |
| Adjusted R^2^ | 0.011 | 0.0003 | 0.019 | -0.004 | | 0.040 | | 0.308 | | 0.313 |

*Notes:* OLS estimates with robust standard errors in parentheses. The omitted category is the treatment “**Control Intervention**” (i.e., no intervention). Control variables include fear of punishment, reward expectation, owner’s responsibility, cost of reporting a wallet, and the perception that safekeeping a wallet without reporting it is moral. Institutions fixed effect was also included. Significance level: ***** *P* < 0.0001; **** *P* < 0.001, *** *P* < 0.01, ** *P* < 0.05, * *P* < 0.10.

#

# **Table S5: Estimated treatment effects on civic honesty using logistic regression (Field Experiment)**

|  | Dependent variable: | |
| --- | --- | --- |
|  | Call response rates | |
|  | (1) | (2) |
| Altruistic concern intervention | -0.081  (0.232) | -0.048  (0.239) |
| Civic responsibility intervention | 0.133  (0.238) | 0.225  (0.246) |
| Safety assurance intervention | -0.667***  (0.230) | -0.642***  (0.237) |
| Male |  | 0.036  (0.192) |
| Age 40+ |  | -0.547***  (0.188) |
| Mobile |  | 0.136  (0.209) |
| Coworkers |  | -0.016  (0.186) |
| Other bystanders |  | -0.115  (0.191) |
| Busyness |  | -0.009  (0.058) |
| Understood situation |  | 0.013  (0.074) |
| Security camera |  | -0.186  (0.204) |
| Security guard |  | 0.324  (0.211) |
| City Fixed Effect | Yes | Yes |
| Institution Fixed Effect | Yes | Yes |
| Constant | 0.754***  (0.226) | 0.873*  (0.489) |
| *N* | 658 | 658 |

*Notes*: The call response rates were analyzed using a logistic regression model. Estimates with standard error in parentheses. The omitted category is the treatment “Control condition” (i.e., no intervention). Control variables include a recipient’s age, gender, the presence of a recipient’s mobile phone, coworkers, other bystanders, whether a recipient understood the situation communicated, whether a recipient was busy, the presence of security camera and security guard. City and Institutions fixed effect were also included. Significance levels: * *P* < 0.10, ** *P* < 0.05, *** *P* < 0.01.

# **Table S6: Estimated treatment effects on civic honesty using linear regression (Field Experiment)**

|  | Dependent variable: | |
| --- | --- | --- |
|  | Call response rates | |
|  | (1) | (2) |
| Altruistic concern intervention | -2.082  (5.372) | -1.089  (5.411) |
| Civic responsibility intervention | 2.547  (5.360) | 5.120  (5.439) |
| Safety assurance intervention | -16.018***  (5.445) | -14.851***  (5.513) |
| Male |  | 3.574  (4.104) |
| Age 40+ |  | -15.736****  (4.240) |
| Mobile |  | 2.736  (4.743) |
| Coworkers |  | -0.242  (4.306) |
| Other bystanders |  | -2.765  (4.406) |
| Busyness |  | -0.123  (1.339) |
| Understood situation |  | 0.656  (1.744) |
| Security camera |  | -2.902  (4.583) |
| Security guard |  | 11.429***  (4.385) |
| City Fixed Effect | Yes | Yes |
| Institution Fixed Effect | Yes | Yes |
| Constant | 63.380****  (5.916) | 63.492****  (11.343) |
| Observations | 658 | 658 |
| Adjusted R^2^ | 0.019 | 0.062 |

*Notes:* OLS estimates with robust standard errors in parentheses. The dependent variable has a value 100 if a wallet was reported, and 0 otherwise. The omitted category is the treatment “Control condition” (i.e., no intervention). Control variables include a recipient’s age, gender, the presence of a recipient’s mobile phone, coworkers, other bystanders, whether a recipient understood the situation communicated, whether a recipient was busy, the presence of security camera and security guard. City and Institutions fixed effect were also included. Significance level: **** *P* < 0.001, *** *P* < 0.01, ** *P* < 0.05, * *P* < 0.10.

# **Table S7: Descriptive statistics and randomization check for Field Experiment**

|  | **Civic responsibility intervention** | | **Altruistic concern intervention** | | **Safety assurance intervention** | | **Control**  **(No intervention)** | | **Total sample** | |  |
| --- | --- | --- | --- | --- | --- | --- | --- | --- | --- | --- | --- |
|  | mean | *SD* | mean | *SD* | mean | *SD* | mean | *SD* | mean | *SD* | *P*-value |
| Age ≥ 40 | 0.35 | 0.48 | 0.35 | 0.48 | 0.32 | 0.47 | 0.31 | 0.46 | 0.33 | 0.47 | 0.888 |
| Male | 0.42 | 0.49 | 0.46 | 0.50 | 0.49 | 0.50 | 0.41 | 0.49 | 0.45 | 0.50 | 0.436 |
| Mobile | 0.22 | 0.42 | 0.28 | 0.45 | 0.25 | 0.43 | 0.26 | 0.44 | 0.25 | 0.44 | 0.658 |
| Coworkers | 0.59 | 0.49 | 0.62 | 0.49 | 0.66 | 0.48 | 0.71 | 0.45 | 0.65 | 0.48 | 0.113 |
| Other Bystanders | 0.40 | 0.49 | 0.38 | 0.49 | 0.41 | 0.49 | 0.41 | 0.49 | 0.40 | 0.49 | 0.911 |
| Local Resident | 0.32 | 0.47 | 0.31 | 0.46 | 0.32 | 0.47 | 0.31 | 0.46 | 0.31 | 0.46 | 0.988 |
| Understood (0 – 6) | 4.92 | 1.14 | 4.93 | 1.14 | 4.89 | 1.18 | 4.94 | 1.20 | 4.92 | 1.16 | 0.982 |
| Busyness (0 – 6) | 1.93 | 1.65 | 1.86 | 1.53 | 1.75 | 1.57 | 2.03 | 1.64 | 1.89 | 1.60 | 0.448 |
| Security Camera | 0.74 | 0.44 | 0.72 | 0.45 | 0.70 | 0.46 | 0.72 | 0.45 | 0.72 | 0.45 | 0.847 |
| Security Guard | 0.31 | 0.47 | 0.41 | 0.49 | 0.34 | 0.48 | 0.40 | 0.49 | 0.37 | 0.48 | 0.200 |
| Cultural Attraction | 0.30 | 0.46 | 0.31 | 0.46 | 0.30 | 0.46 | 0.29 | 0.45 | 0.30 | 0.46 | 0.981 |
| Hotel | 0.29 | 0.45 | 0.30 | 0.46 | 0.28 | 0.45 | 0.30 | 0.46 | 0.29 | 0.46 | 0.987 |
| Pharmacy | 0.31 | 0.47 | 0.30 | 0.46 | 0.31 | 0.47 | 0.30 | 0.46 | 0.31 | 0.46 | 0.978 |
| Public Office | 0.09 | 0.29 | 0.10 | 0.30 | 0.10 | 0.30 | 0.11 | 0.31 | 0.10 | 0.30 | 0.970 |

*Notes:* “Age ≥ 40” was coded as 1 if a recipient was perceived to be 40 years old or older, and 0 otherwise. “Male” was coded as 1 if the recipient was male and 0 otherwise. “Mobile” was coded as 1 if a recipient was carrying a mobile phone, and 0 otherwise. “Coworkers” was coded as 1 if coworkers of a recipient were involved in the interaction, and 0 otherwise. “Other bystanders” was coded as 1 if observers were present during the interaction, and 0 otherwise. “Local resident” was coded as 1 if a recipient was perceived to be a local resident, and 0 otherwise. “Understood” was a rating on the extent to which a recipient was perceived to be clear about the wallet situation communicated (0 = not at all to 6 = very clear). “Busyness” was a rating on the extent to which a recipient was perceived to be busy before the wallet was turned in (0 = not at all to 6 = very busy). “Security camera” was coded as 1 when security camera was present in the institution, and 0 otherwise. “Security guard” was coded as 1 when security guard was present, and 0 otherwise. “Cultural attraction,” “Hotel,” “Pharmacy,” “Public Office,” were the four types of institutions for drop-off locations. The last column shows P-values for the null hypotheses of perfect randomization (we used χ² tests for the categorial variables and ANOVA for the two continuous variables, “Understood,” and “Busyness.”).

1. The amount of money in the Money and BigMoney conditions is based on those in Cohn et al. and had been adjusted for inflation. [↑](#footnote-ref-1)
2. Participants also reported the extent to which they were worried that the QR link contained virus and would be accused of having pocketed money from the lost wallet. We did not observe consistently significant effects across conditions and across the two online experiments. For ease of exposition, we will not discuss these analyses in detail. [↑](#footnote-ref-2)
3. Robust standard errors are reported for all linear regression analyses in all studies. [↑](#footnote-ref-3)
